# Innate activation of human primary epithelial cells broadens the host response to *Mycobacterium tuberculosis* in the airways

**DOI:** 10.1371/journal.ppat.1006577

**Published:** 2017-09-01

**Authors:** Ann-Kathrin Reuschl, Michael R. Edwards, Robert Parker, David W. Connell, Long Hoang, Alice Halliday, Hannah Jarvis, Nazneen Siddiqui, Corrina Wright, Samuel Bremang, Sandra M. Newton, Peter Beverley, Robin J. Shattock, Onn Min Kon, Ajit Lalvani

**Affiliations:** 1 Tuberculosis Research Centre, National Heart and Lung Institute, Imperial College London, St Mary’s Campus, London, United Kingdom; 2 Department of Cytopathology, Imperial College London, St Mary’s Hospital, Imperial College NHS Trust, London, United Kingdom; 3 Respiratory Medicine, National Heart and Lung Institute, Imperial College London, St Mary’s Campus, Norfolk Place, London, United Kingdom; 4 Section of Paediatrics, Department of Medicine, St Mary’s Campus, Imperial College, London, United Kingdom; 5 Department of Medicine, Imperial College London, St Mary’s Campus, London, United Kingdom; University of Washington, UNITED STATES

## Abstract

Early events in the human airways determining whether exposure to *Mycobacterium tuberculosis* (Mtb) results in acquisition of infection are poorly understood. Epithelial cells are the dominant cell type in the lungs, but little is known about their role in tuberculosis. We hypothesised that human primary airway epithelial cells are part of the first line of defense against Mtb-infection and contribute to the protective host response in the human respiratory tract. We modelled these early airway-interactions with human primary bronchial epithelial cells (PBECs) and alveolar macrophages. By combining *in vitro* infection and transwell co-culture models with a global transcriptomic approach, we identified PBECs to be inert to direct Mtb-infection, yet to be potent responders within an Mtb-activated immune network, mediated by IL1β and type I interferon (IFN). Activation of PBECs by Mtb-infected alveolar macrophages and monocytes increased expression of known and novel antimycobacterial peptides, defensins and S100-family members and epithelial-myeloid interactions further shaped the immunological environment during Mtb-infection by promoting neutrophil influx. This is the first in depth analysis of the primary epithelial response to infection and offers new insights into their emerging role in tuberculosis through complementing and amplifying responses to Mtb.

## Author summary

*Mycobacterium tuberculosis* (Mtb) is the causative agent of tuberculosis, which remains a major public health burden today. In the majority of cases, infection is acquired by inhalation of aerosolised bacteria. Mtb is thought to target alveolar macrophages in the lower airways to establish infection. However, the cells predominantly lining the respiratory tract are epithelial cells and thus are likely crucial during the early host-pathogen interactions. We recovered primary human bronchial epithelial cells from healthy volunteers to assess their global transcriptomic response to direct Mtb-exposure and exposure to Mtb-infected myeloid cells. Our analysis revealed that, while being inert to direct Mtb-infection, epithelial cells were highly responsive to soluble mediators released by infected macrophages. The epithelial response induced by this cellular cross-talk, promoted neutrophil influx *in vitro* as well as the increase of antimycobaterial host responses. Our data provide novel and unexpected insights into the role of the primary human airway epithelium and define a non-redundant role for epithelial cells in shaping the local immunological environment at the site of initial Mtb infection.

## Introduction

The first interactions between *Mycobacterium tuberculosis* (Mtb) and its human host occur in the lungs after inhalation of aerosolised bacteria. Approximately fifty percent of individuals exposed to Mtb remain uninfected [1] and host-determinants have been associated with this resistance to or clearance of infection in humans [2]. The absence of a detectable peripheral adaptive immune response in resistant individuals suggests that it is mediated by the local innate immune system and that the respiratory mucosa plays a role in determining the outcome of exposure [3]. It is assumed that the primary target of Mtb, an intracellular pathogen, is the alveolar macrophage in the lower airways. However, the majority of cells in the airway lining are epithelial which constitute a surface of approximately 70 m^2^ [4] and are thus likely to be the first point of contact for Mtb in the human host.

Epithelial cells are known to significantly contribute to the immune responses in the lungs and can sense intra- and extracellular pathogens, such as viruses and bacteria, via a wide range of pattern recognition receptors (PRR) [5]. In contrast to this, their responses in tuberculosis (TB) are poorly defined and little investigated, which presents a surprising knowledge gap.

Recognition by, or infection of, epithelial cells in the human airways may shape early host responses to the bacilli and act as an adjunct to the immune response against infections. Work on human airway epithelial-like cell lines and murine airway epithelial cells has identified potential mechanisms of recognition and response to Mtb-infection, including chemokine release and antimicrobial action [6–8]. However, the response of primary human airway epithelial cells to Mtb infection has barely been investigated and has so far mainly concentrated on isolated pathways rather than the global epithelial response to Mtb-infection [9–11]. The need to investigate the interactions of Mtb with primary human airway epithelial cells is further underscored by detection of Mtb in these cells during infections *in vivo*. Previous studies reported that in animal models of tuberculosis infection the bronchial and alveolar epithelium is infected after instillation with Mtb [12,13] and Mtb DNA was detected in human epithelial cells of histologically normal lung tissue in healthy latently infected individuals [14].

In this study, we hypothesised that human primary airway epithelial cells contribute to the protective early host response to Mtb-infection in the human respiratory tract. To test this, we interrogated the direct response of epithelial cells to Mtb as well as indirect effects mediated via an immune-network comprising infected myeloid cells, by measuring the global epithelial transcriptome. Through *in vitro* infection and transwell co-culture models, we identified primary bronchial epithelial cells to be inert to direct Mtb-infection, yet to be potent responders within an Mtb-activated immune network, mediated by IL1 β and type I IFN, with the capacity to shape the local antimicrobial and immunological environment.

## Results

### Bronchial epithelial cells dominate the airway lining and are resistant to Mtb challenge *in vitro*

**Fig 1:**
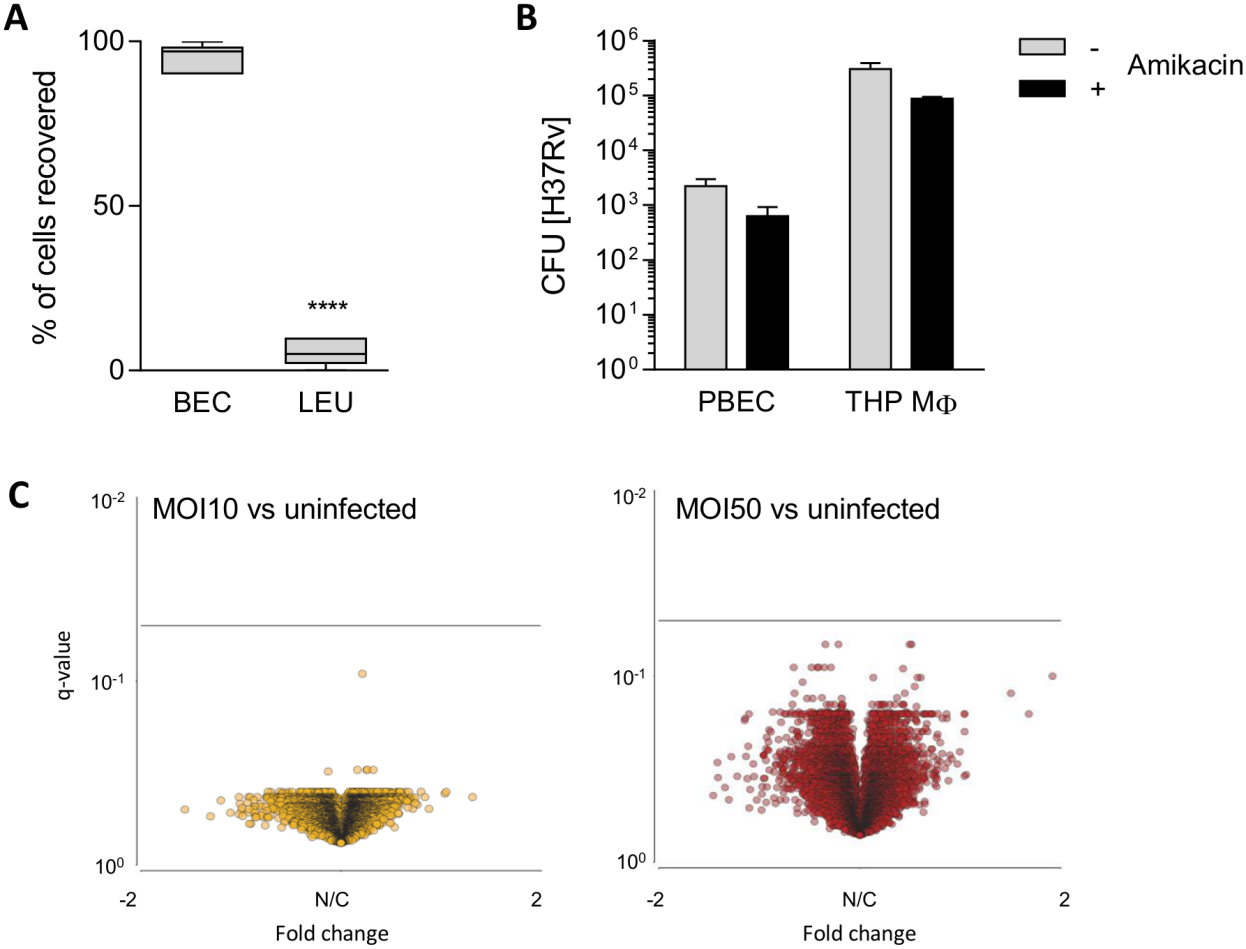
Epithelial cells are the major subset in the airway lining and are not permissive to Mtb infection. (A) Presence of total bronchial epithelial cells (BECs) and leukocytes (LEU) was measured *ex vivo* by differential cell counts of bronchial brushings derived from the airway lining. Subsets are shown as % of total cells recovered from healthy volunteers (n = 17). Wilcoxon signed rank test was used to compare groups. Boxplots show median and range. **** p<0.0001. (B) PBECs (n = 4) and THP-1 MΦ (mean ± SD of 2 independent experiments) were infected with Mtb H37Rv for 24h. To remove extracellular bacteria, 200 μg/ml amikacin was added for an additional 2h where indicated. Mtb bacilli were enumerated by colony forming units (CFU). (C) Affymetrix HTA2.0 arrays were performed on PBECs (uninfected, MOI10 or MOI50, 24h) from 4 donors. The log_2_ fold change compared to unstimulated PBECs and the associated FDR-adjusted q-value are shown in volcano plots for MOI10 (left) and MOI50 (right). The solid black lines intersect at q-value of 0.05.

In healthy human airways, epithelial cells were the major cell type lining the respiratory tract and harboured less than 10% of leukocyte subsets (Fig 1A), of which macrophages, lymphocytes and neutrophils contributed on average 86.4%, 12.5% and 1.1%, respectively. The abundance of epithelial cells increases the likelihood that Mtb interacts directly with the epithelial lining after inhalation of aerosols. To assess whether human airway epithelial cells are permissive to intracellular Mtb-infection, primary bronchial epithelial cells (PBECs) recovered from bronchial brushes were expanded *in vitro* and infected with Mtb. The total cell-associated mycobacterial burden, after amikacin treatment, was over two logs less in PBECs than the Mtb-burden in THP-1 macrophages after 24h (Fig 1B). The decreased association of Mtb with PBECs was confirmed by microscopy in the presence of an increased multiplicity of infection (MOI) (S1 Fig). The minimal level of epithelial infection was reflected by a lack of transcriptomic changes in PBECs after exposure to different MOIs of Mtb for 24h (Fig 1C). Microarray analysis revealed no significant differences in gene expression after statistical adjustment for multiple comparisons. Since PBECs were poor direct responders to Mtb exposure and infection, yet macrophages were readily infected, we reasoned that the role of PBECs during the early host response to Mtb might be as part of the Mtb-activated immune network with myeloid cells as the primary replicative niche for Mtb.

### PBECs express antimicrobial peptides and immune mediators in response to Mtb-induced myeloid activation

**Fig 2:**
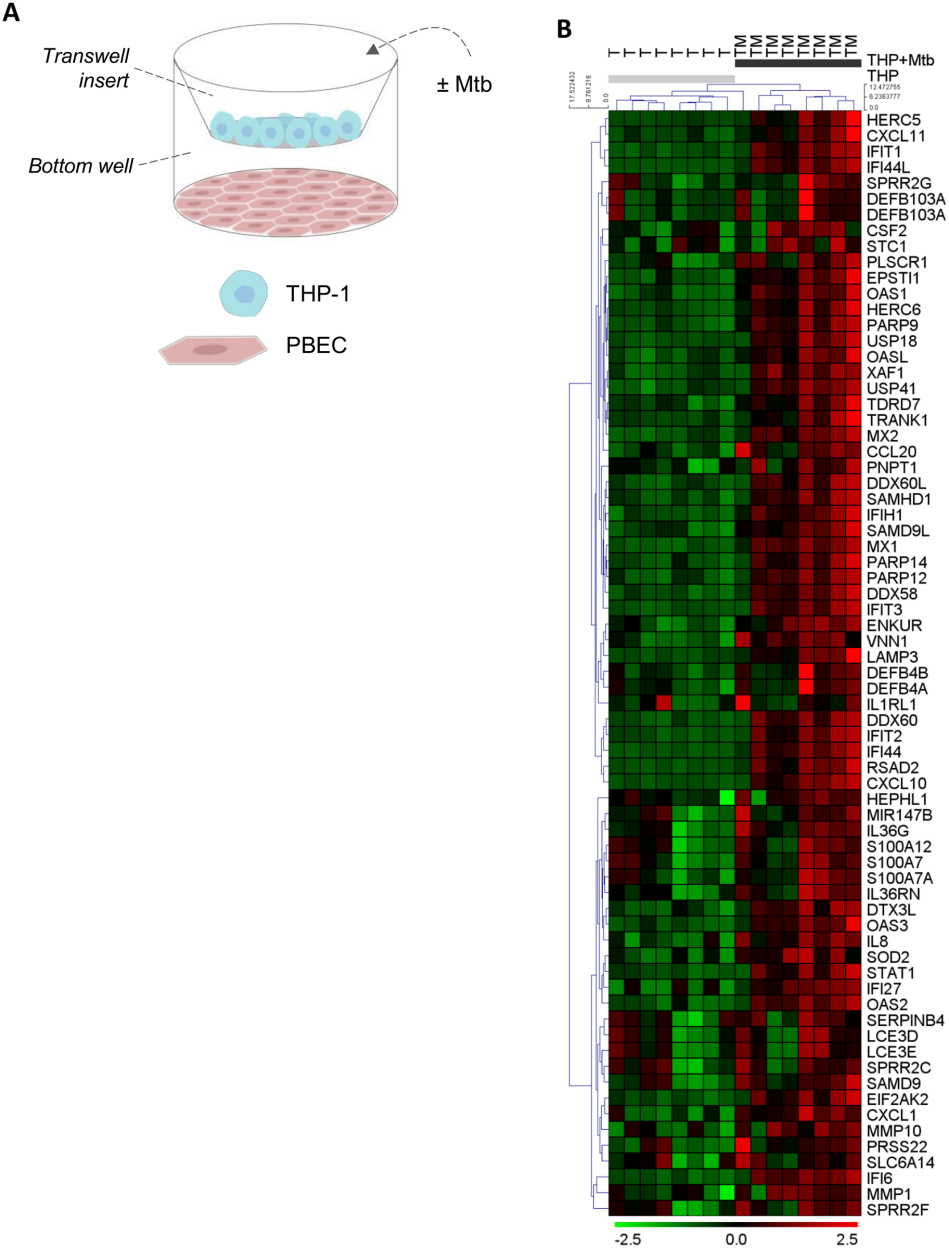
Whole transcriptome analysis of PBECs exposed to Mtb-infected myeloid cells. (A) To interrogate the effect of myeloid-epithelial cross-talk during Mtb infection, a transwell co-culture system was established. Separated by a 0.4 μm pore-sized transwell membrane to allow exchange of soluble mediator only, PBECs were seeded in the bottom well of a tissue culture plate and THP-1 cells were added into the transwell insert. Cells were infected as indicated. (B) In the transwell co-culture model, PBECs from eight donors were exposed to uninfected (THP, T) or Mtb-infected THP-1 (THP + Mtb, TM) cells for 24h. Affymetrix HTA2.0 microarrays were performed on PBECs. Hierarchical clustering of all significantly differentially expressed genes at a fold change of > 1.5 and a q-value < 5% was performed using average linkage and Euclidean distance. Expression range from low (green) to high (red).

**Fig 3:**
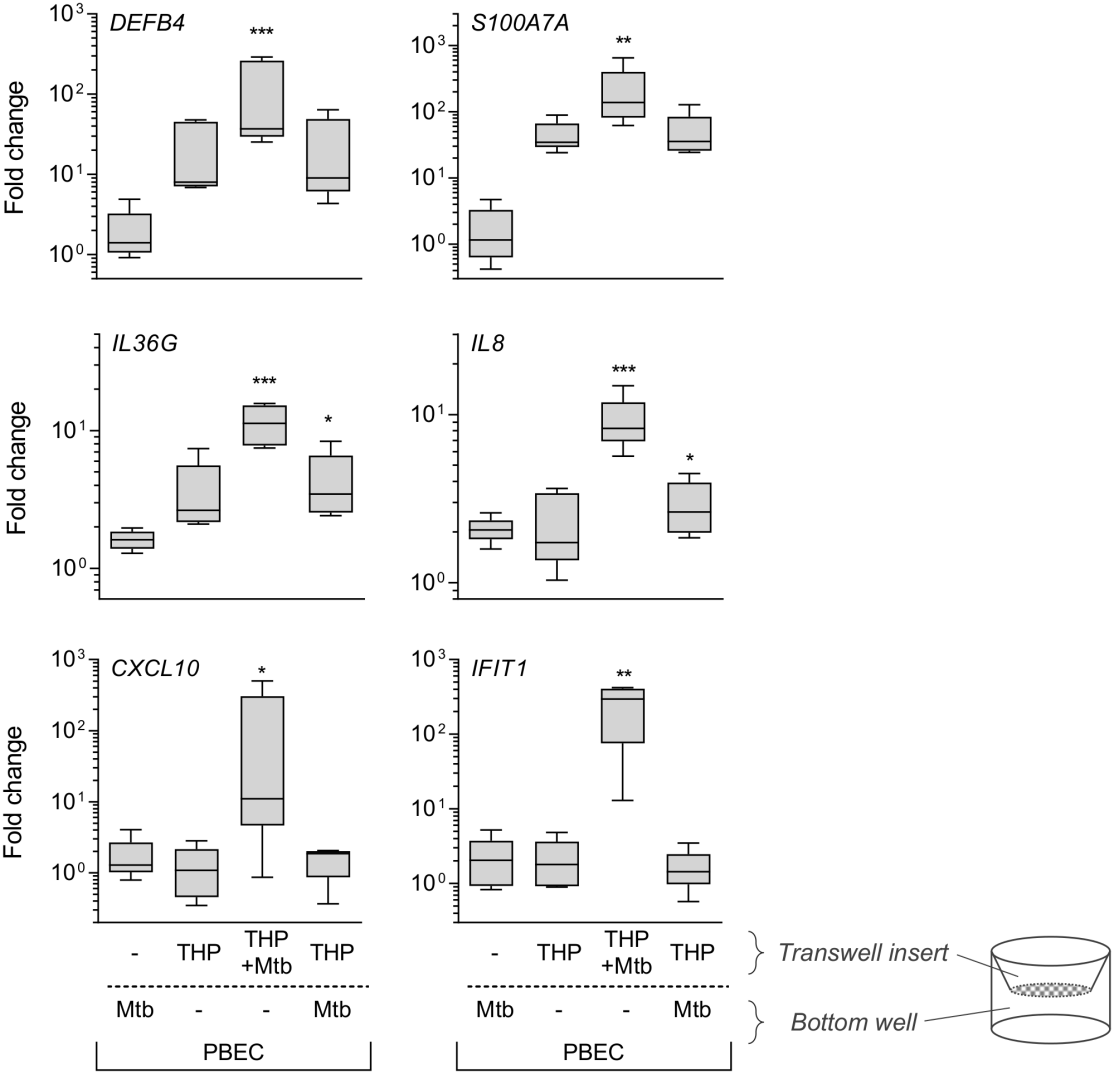
PBEC gene expression in response to infection-driven inflammation is dependent on direct interaction between myeloid cells and Mtb. In the transwell model, PBECs were exposed to THP-1 cells or Mtb H37Rv bacilli (MOI5) for 24h as indicated. Gene expression in PBECs was measured by RT-PCR and is shown as fold change over unstimulated PBECs (n = 5). Friedman test with Dunn’s post-test was used to compare expression with unstimulated controls (fold change = 1, not displayed). Boxplots show median and range. * p<0.05; **, p<0.01; ***, p<0.001; ****, p<0.0001.

After infection with Mtb, macrophages, the largest leukocyte subset in the healthy airways [15,16], can mount a pro-inflammatory response. To investigate if PBECs, whilst inert to direct interaction with Mtb bacilli, are affected by these events, we established a contact-independent co-culture system to measure the global epithelial response to myeloid-derived Mtb-induced mediators (Fig 2A). THP-1 monocytic cells were used to mimic the myeloid compartment in the airways and were infected with Mtb or remained uninfected. Microarrays were performed on PBECs derived from eight healthy donors exposed to these two conditions to model cross-talk in the healthy and infected lungs (Fig 2B). Four-hundred-twenty-eight probesets representing 375 genes were differentially expressed in PBECs exposed to Mtb-infected THP-1 cells compared to uninfected THP-1 cells (q-value<5%). Seventy of these were at least 1.5 fold differentially expressed (Fig 2B, see S1 Table for full list). The identified expression signature included cytokines and antimicrobial peptides and 6 of these genes were selected for independent validation by RT-PCR (Fig 3): *DEFB4* and *S100A7A*, which encode known antimicrobial peptides β-defensin 2 (hBD2) and koebnerisin [17,18]; *IL36G* which is an emerging IL1-family member and may be beneficial during tuberculosis infection [19] the chemokines *IL8* and C-X-C motif ligand (*CXCL*) 10; and two known type I IFN inducible targets interferon-induced protein with tetratricopeptide repeats (*IFIT*) 1 and interferon-induced protein (*IFI*) 44. The RT-PCR validation experiment confirmed the gene expression patterns identified by microarray and showed that the signature was strongly dependent on exposure to infected THP-1 cells and was not inducible by direct infection of PBECs with Mtb, which confirmed the epithelial inertia to Mtb. While myeloid infection with virulent Mtb had profound effects on epithelial gene expression, non-pathogenic *M*. *bovis* BCG infection of THP-1 cells at an MOI of 10 only increased epithelial *CXCL10* expression to 9 or 19% of the gene expression induced by Mtb in two independent experiments, respectively.

**Fig 4:**
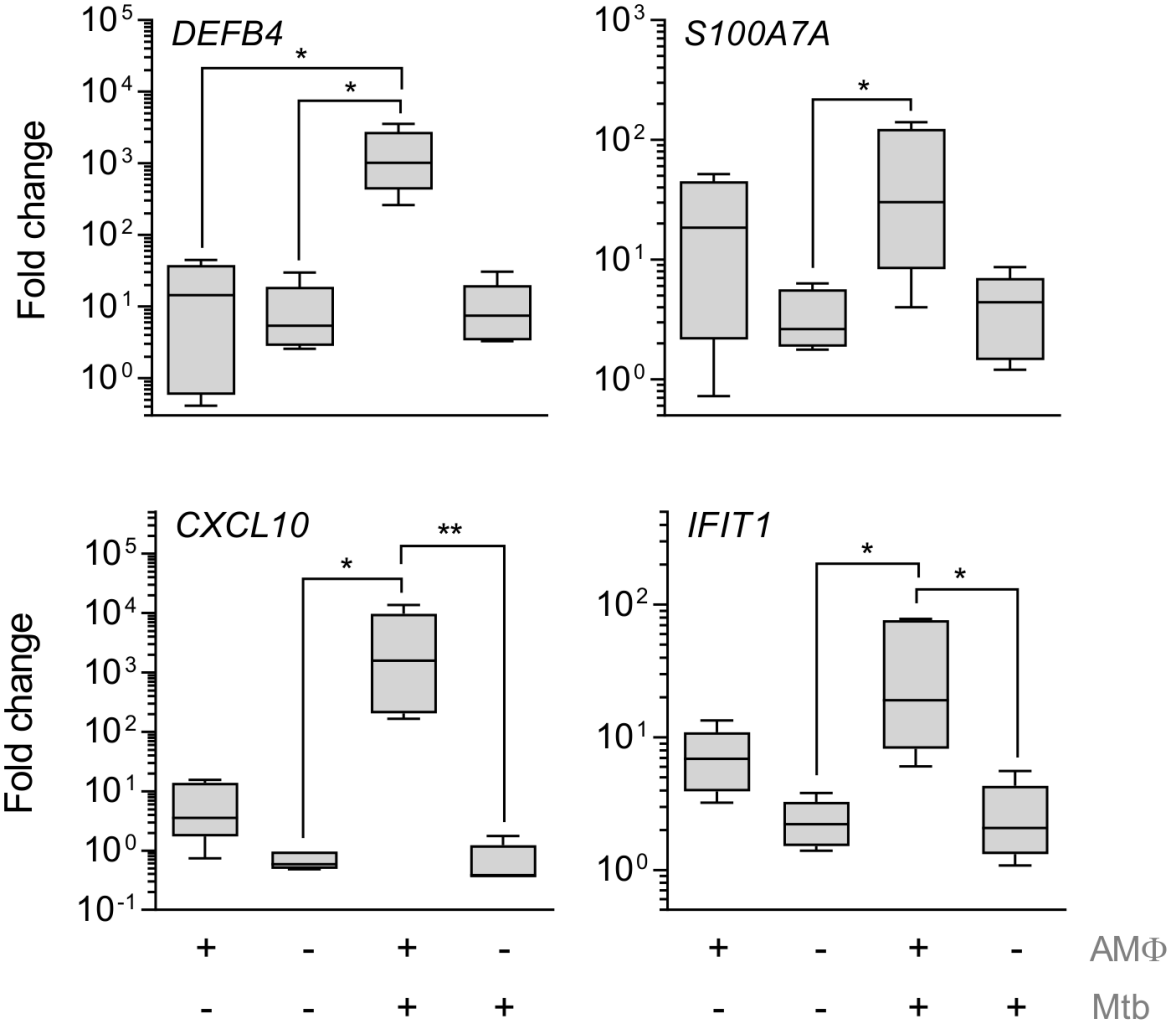
Mtb-infected alveolar macrophages drives gene expression in PBECs. Cell-free conditioned medium from Mtb-infected and uninfected AMΦ, alongside the appropriate culture medium controls was added to PBECs. After 24h gene expression was measured by RT-PCR. Gene expression is shown as fold change over unstimulated PBECs (n = 5). Friedman test with Dunn’s post-test was used to compare stimulations. Boxplots show median and range. * p<0.05; **, p<0.01 or the exact p-value are given.

THP-1 cells are a valuable model for *in vitro* studies of myeloid responses to Mtb, and we confirmed that our model reflected responses in the lungs through the use of primary alveolar macrophages (AMΦ). For this, PBECs were exposed to infected primary AMΦs from independent donors in co-culture (n = 2, S2 Fig), which confirmed increased expression of *DEFB4*, *S100A7A*, *CXCL10* and *IFIT1*. Transwell co-culture with primary macrophages could only be performed twice and cell-free supernatant of infected and uninfected AMΦs was used to confirm expression patterns in depth (Fig 4). Soluble mediators secreted by AMΦs induced similar transcriptomic responses as observed when THP-1 cells were used. Thus, the epithelial response to Mtb-induced inflammation we observed in our model mimicked paracrine AMΦ-epithelial signalling that may occur early after exposure of the human airways to Mtb.

### PBEC responses to Mtb-induced myeloid inflammation are driven discretely by IL1β and type I IFN

**Fig 5:**
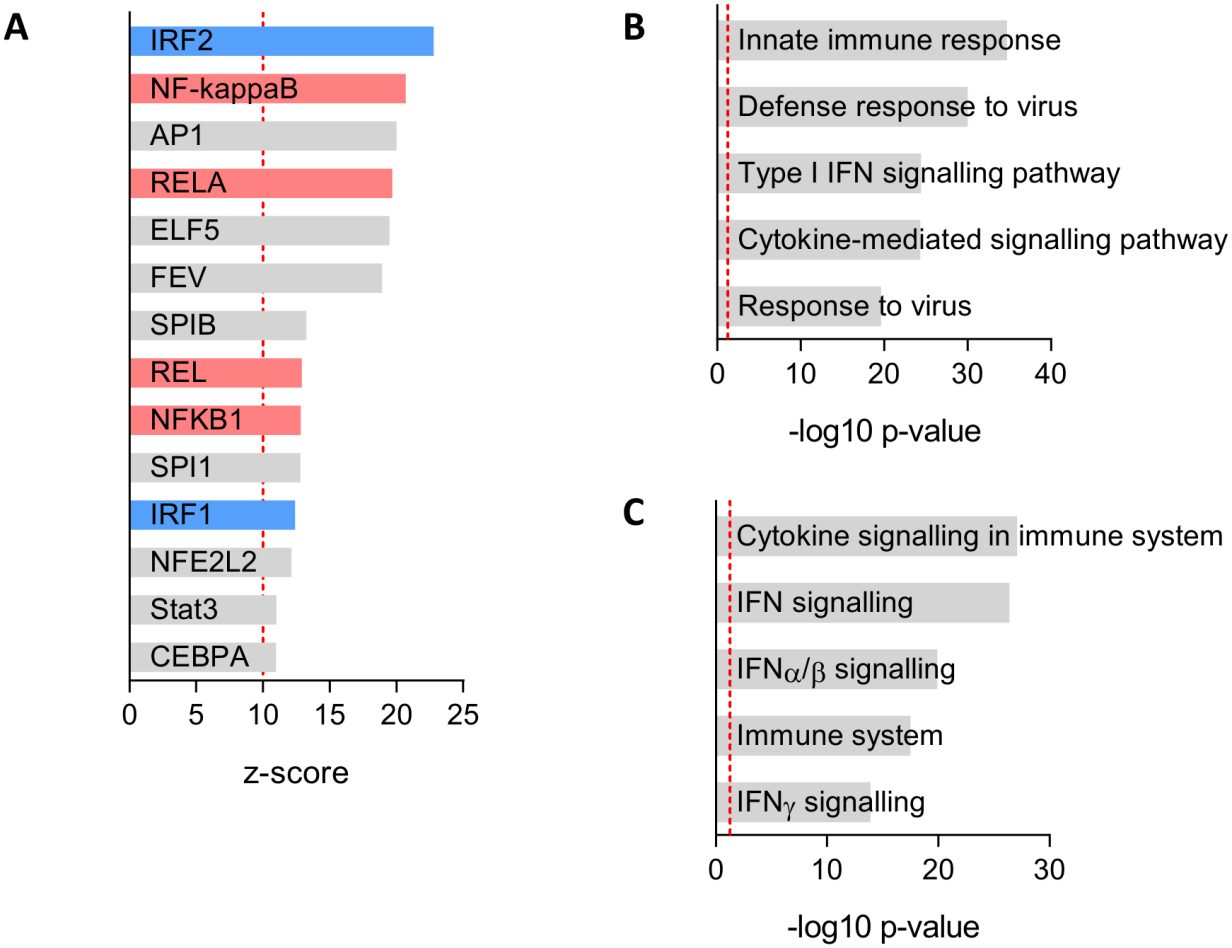
PBEC responses to Mtb-driven inflammation are enriched for pro-inflammatory and interferon-mediated signalling. All DE genes at a q-value < 5% were used for statistical over-representation analysis (ORA) by InnateDB and oPOSSUM. The most significant (A) Transcription Factor Binding Sites (TFBS), (B) Pathways and (C) Gene ontology terms are shown. Red dashed line indicates a z-score of 10 or a corrected p-value of 0.05. For (A), IRF- and NFκB-family members are highlighted in blue and red respectively.

To identify the Mtb-induced inflammatory signals which drove gene expression in PBECs, we performed transcription factor binding site (TFBS) enrichment analysis with oPossum which revealed that the epithelial expression signature was associated with nuclear factor kappa-light-chain-enhancer of activated B cells (NFκB), including RelA and the p105 subunit (NFKB1), and interferon regulatory factor (IRF)-family members 1 and 2 (Fig 5A). Exposure of PBECs to myeloid Mtb-infection furthermore induced pathways involved in immune response signalling, including interferon-driven pathways (Fig 5B). Similarly, gene ontology was enriched for terms associated with cytokine and IFN signalling (Fig 5C).

**Fig 6:**
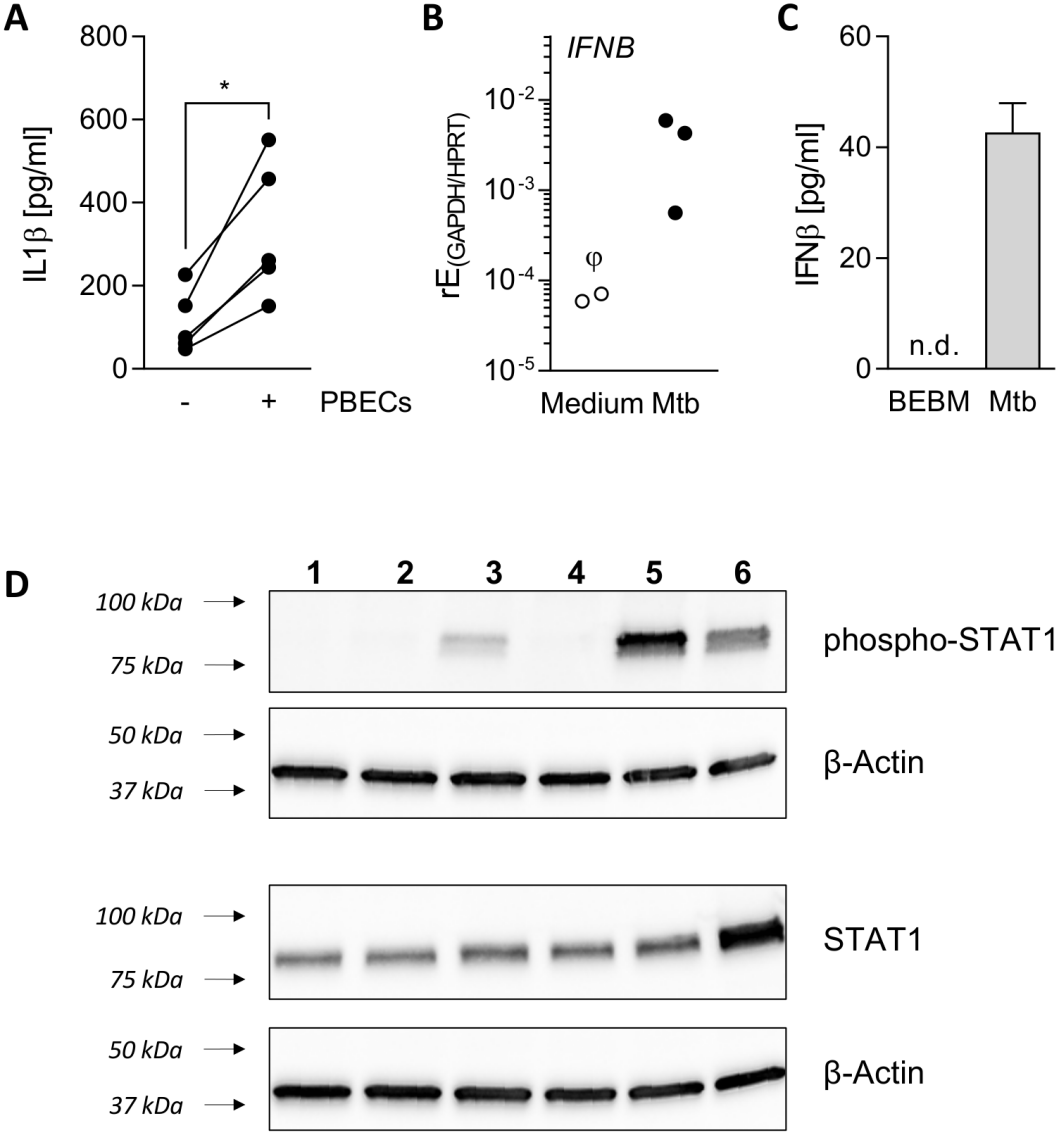
IL1β and IFN signalling are activated during Mtb-mediated inflammation. (A) In co-culture, IL1β release from Mtb-infected THP-1 cells (MOI5) was measured in the presence or absence of PBECs at 24h (n = 5). (B) AMФs from three donors were infected with Mtb H37Rv (MOI5) for 24h. *IFNB* expression was measured by RT-PCR and is shown as relative expression (rE) over *GAPDH* and *HPRT*. ϕ, not detected in one donor. (C) IFNβ release by THP-1 cells after 24h of infection was measured by ELISA. Mean ± SD are shown (n = 3). (D) Western blot for STAT1 and phospho-STAT1 on PBECs stimulated for 24h with medium (1), transwell co-culture with uninfected (THP) (2) or Mtb-infected (THP + Mtb) (3) THP-1 cells or stimulated with 10 ng/ml recombinant IL1β (5h, 4) or IFNβ (5h, 5 and 24h, 6). Shown is one representative blot of three independent experiments. One-tailed Wilcoxon signed rank test was used to compare groups in (A) and (B). n.d., not detected; *, p<0.05.

It is well known that macrophages secrete several inflammatory mediators in response to Mtb-infection, including IL1β and IFNβ [20–22]. We found that, IL1β levels in culture supernatants were over threefold enhanced when infected THP-1 cells were co-cultured with PBECs (Fig 6A). A similar increase was not observed to the same extent when THP-1 cells were infected with BCG in the presence of PBECs, which resulted in a release of only 28.9–81.2 pg/ml IL1β during co-culture in three independent experiments. IFN signalling was indicated by the pathway enrichment analysis and we confirmed IFN β expression and release from myeloid cells to identify whether type I IFNs may have been involved in epithelial activation. We detected increases in gene expression and protein secretion during Mtb-infection in AMΦ and THP-1 cells(Fig 6B and 6C). IFNβ induction co-incided with phosphorylation of STAT1, a downstream mediator of type I IFN-signalling, in PBECs exposed to Mtb-infected THP-1 cells (Fig 6D). *IFNB* was not induced in PBECs during co-culture (S3A Fig) and neither IFNγ nor IFNλ could be detected by ELISA in culture supernatants of THP-1 cells 24h after Mtb-infection (S3B Fig).

**Fig 7:**
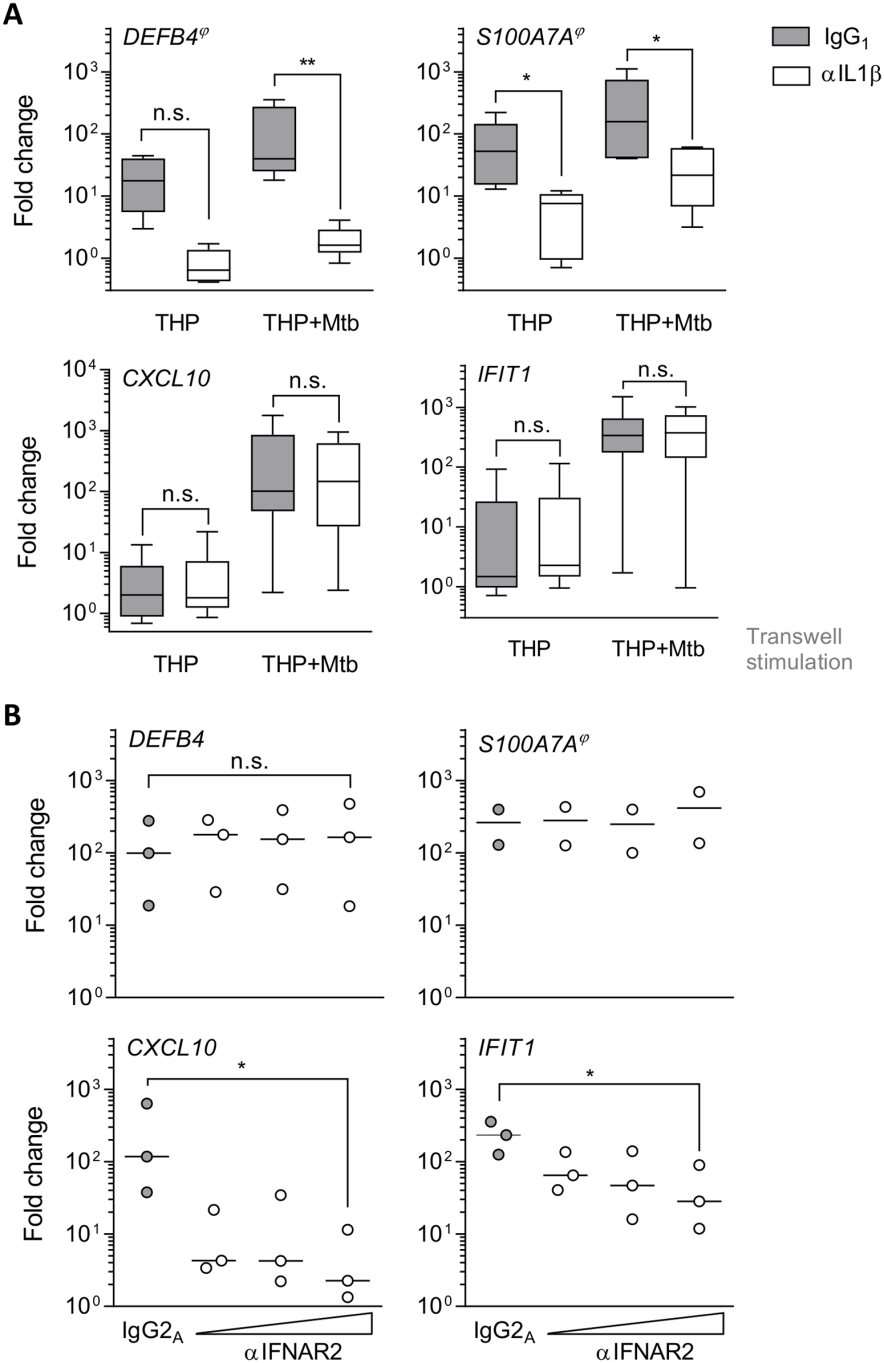
Mtb-driven inflammation activates PBECs in an IL1β and type I IFN signalling dependent manner. (A) PBECs were co-cultured with infected or uninfected THP-1 cells in the presence of αL1β or IgG_1_ (isotype control) as indicated. After 24h, gene expression was measured by RT-PCR. Expression is shown as fold change over unstimulated (n = 6). Boxplots show median and range. (B) PBECs were exposed to Mtb-infected THP-1 cells (MOI5) in the presence of increasing concentrations of αIFNAR2 (5, 10 and 20 μg/ml) or IgG_2_ (isotype control). After 24h, gene expression was measured by RT-PCR and is shown as fold change over unstimulated (n = 3). Median is shown. Friedman test with Dunn’s post-test was used to compare groups against isotype controls. n.s., not significant; *, p<0.05; **, p<0.01. ϕ, baseline expression not detected in two or one donor for *DEFB4* and *S100A7A*, respectively.

Stimulation with recombinant cytokines confirmed that IL1β and IFNβ were sufficient to induce *DEFB4* and *CXCL10* expression respectively, but not vice versa (S3C Fig). To identify whether IL1 β or type I IFN could in fact mediate the epithelial signature in response to myeloid Mtb-infection, we abrogated each pathway in co-culture. Neutralising IL1β prevented upregulation of AMP genes *DEFB4* and *S100A7A* (Fig 7A) as well as pro-inflammatory cytokines *IL8* and *IL36G* (S3D and S3E Fig). Neutralisation of TNF in Mtb-infected co-cultures or infected THP-1 macrophage monoculture, enhanced the IL1β mediated effects, likely reflecting autocrine activation of THP-1 cells (S4A–S4C Fig). In contrast, genes associated with interferon-signalling, *CXCL10* and *IFIT1*, were unaffected by IL1β neutralisation (Fig 7A) and dependent on IFNAR2 activation (Fig 7B), which mediates type I IFN signalling. Conversely, abrogation of IFNAR2 signalling did not affect the expression of the tested IL1β -dependent genes. Neutralisation of IFNγ had no effect on *CXCL10* expression (S4D Fig).

### PBECs can shape the antimycobacterial and cellular host response to infection during Mtb-driven inflammation

**Fig 8:**
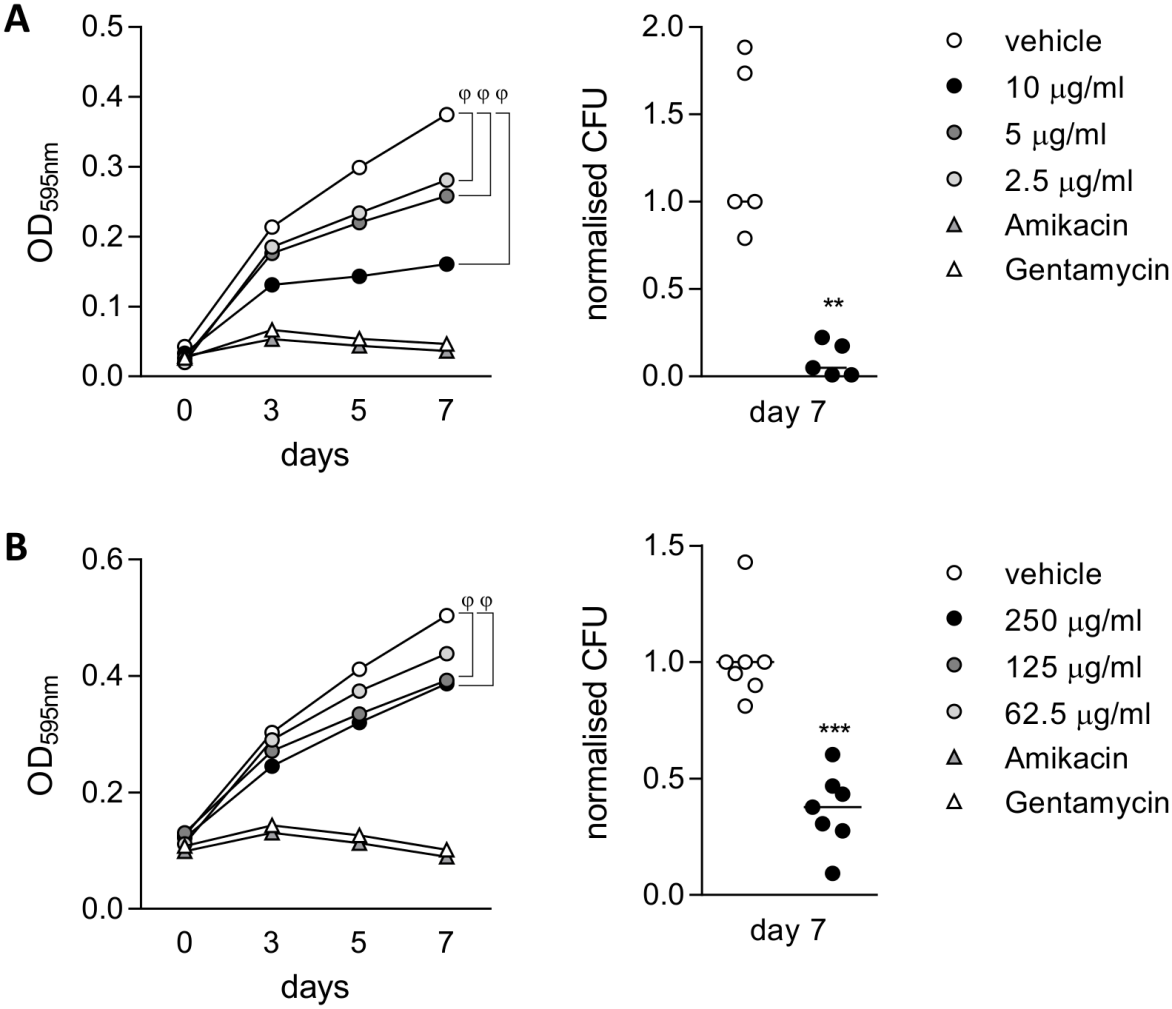
PBECs express antimycobacterial effectors *DEFB4* and *S100A7*. Mtb H37Rv was grown in the presence of the indicated concentrations of recombinant (A) hBD2, (B) psoriasin or vehicle control (veh). Amikacin and gentamycin were used as positive controls at 200 μg/ml and 100 μg/ml respectively. Mtb growth was measured by optical density at 595nm (OD_595nm_) over time. Mean readings of one representative experiment are shown. At day 7, cultures were plated to measure the bacterial burden by CFU (n = 3 representative for two independent H37Rv cultures). Groups were compared against vehicle control at day 7 by one-way ANOVA or Mann-Whitney test (pooled normalised CFU data from three (psoriasin) or two (hBD2) independent experiments are shown). Median is shown. ϕ, p<0.001;**, p<0.01; ***, p<0.001.

*DEFB4* and *S100A7A*, which were strongly expressed in PBECs in response to Mtb-driven inflammation, were not expressed by Mtb-infected THP-1 cells. Mtb-infection also failed to induce *S100A7A* expression in AMΦs, while *DEFB4* induction ranged from 0.84–14.08 fold in three healthy donors. Since the release of AMPs may critically determine the outcome of exposure to Mtb, we tested whether the infection-induced defensins and S100A-family members control free growing Mtb. Human β -defensin 2 (hBD2), which is encoded by *DEFB4*, has been previously described to diminish Mtb growth in a resazurin assay [17] and was a potent inhibitor of growth of Mtb H37Rv (Fig 8A) and clinical strains in our hands (S5A Fig). Because, no bioactive form of S100A7A was available, we used the close homologue S100A7 [23], known as psoriasin, which was also induced in an IL1β-dependent manner in co-culture (S5B and S5C Fig) and reflected the induction pattern observed for *S100A7A* (Fig 3). Psoriasin decreased the median Mtb burden in liquid culture significantly by 62% (Fig 8B).

**Fig 9:**
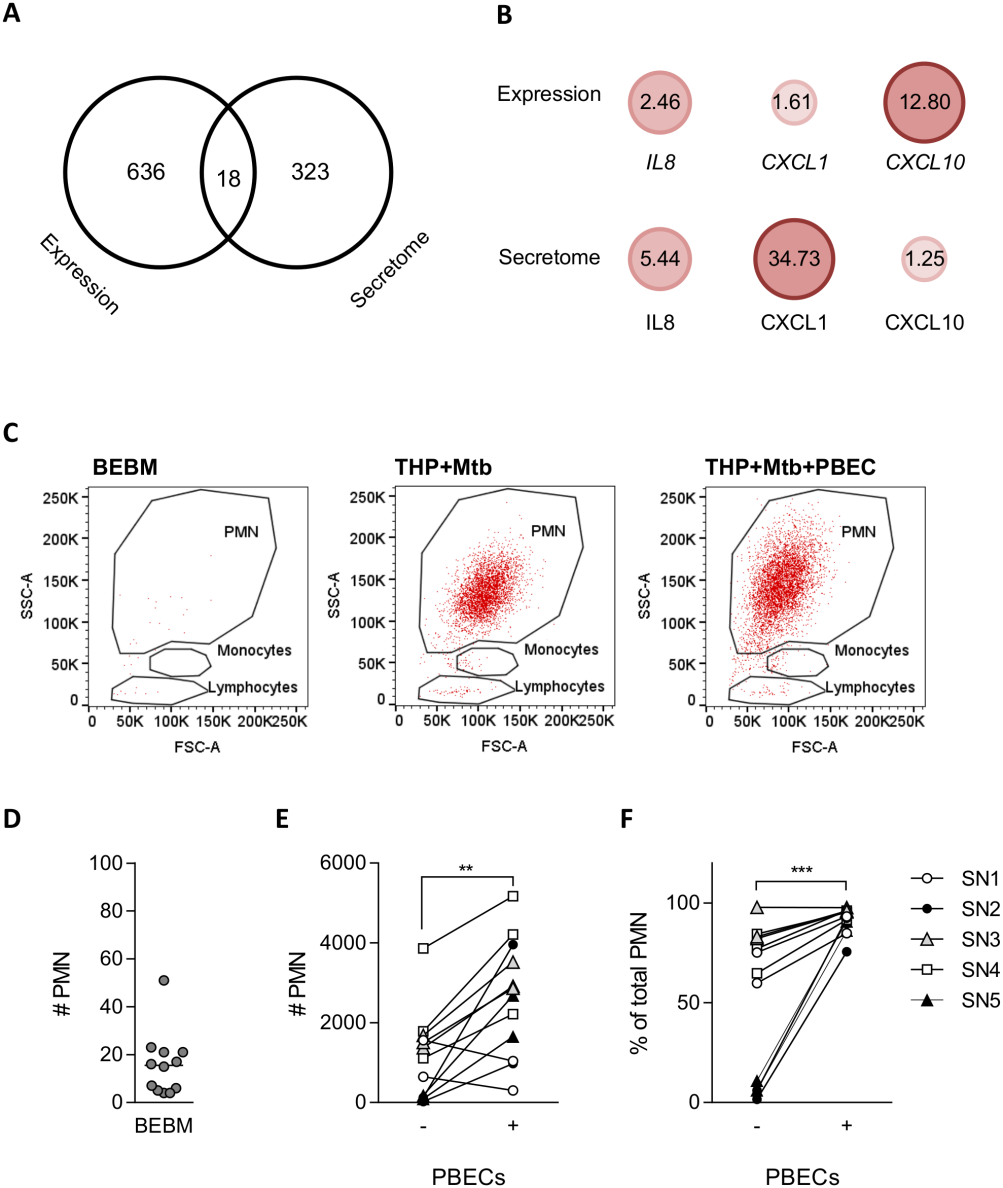
PBECs upregulate chemokines and enhance neutrophil recruitment to the site of Mtb-infection. (A) The Venn diagramme shows the overlap between significantly induced genes in PBECs exposed to Mtb-infected THP-1 cells at 24h (Expression) and significantly increased secreted proteins during infected PBEC-THP-1 co-culture over infected THP-1 monoculture at 48h (Secretome). Three of the overlapping proteins were associated with chemotaxis (GO:0006935) and their fold changes in their respective comparisons are shown in (B). PBLs were added into a transwell insert on top of a culture well containing conditioned medium from Mtb-infected THP-1 cells ± PBECs or medium control (BEBM). Supernatants were generated from five different PBEC donors (SN1-5). After 3 h cells from the insert and the bottom well were collected and enumerated by flowcytometry. (C) Shows representative plots of the gating strategy to define migrated subsets by forward scatter (FSC) and side scatter (SSC). (D) As a background control, migration across the transwell membrane towards medium (BEBM) is shown as total number of recovered PMNs. (E) Number of migrated PMNs towards cell-free conditioned medium derived from Mtb-infected THP-1 cells ± PBECs is shown. (F) Shows PMN migration as % of total cells. Groups were compared by (n = 12). Horizontal bars indicate the median. *, p<0.05; **, p<0.01; ***, p<0.001.

In addition to local responses to Mtb, influx of immune cells to the site of infection can shape the human immune response in the lungs. As part of the myeloid-driven epithelial expression signature identified here, chemokines including *CXCL10* and *IL8* were induced through pro-inflammatory and IFN-driven pathways. Infected myeloid cells secrete chemokines which attract leukocytes [24,25] and in addition to this, constitutive or induced release of mediators by epithelial cells may contribute or amplify timely immune cell recruitment to the site of infection. To address this, we investigated whether the observed gene expression patterns in PBECs were associated with changes in the chemotactic environment during co-culture compared to infected THP-1 monoculture. THP-1 cells were infected with Mtb in the presence or absence of PBECs and cell free culture supernatants were harvested after 48h. The secreted protein content (secretome) was measured through LC-MS. We detected 654 proteins at significantly different levels in co-culture compared to infected THP-1 monocultures (S2 Table). This included increased levels of IL1β in THP-1 PBEC co-culture, which confirmed our previous ELISA data (Fig 6A). To identify which of these proteins were differentially expressed at the gene level in PBECs during co-culture, the gene expression signature was converted into UniProt IDs, which mapped to 341 proteins of which 18 were detected in the transcriptomic and secretome analyses (Fig 9A). Three entities, the leukocyte chemoattractants IL8, CXCL1 and CXCL10, associated with the GO term ‘chemotaxis’ (GO:0006935, AmiGO 2, Gene Ontology Consortium). Myeloid-epithelial cross-talk significantly induced their expression in PBECs and the subsequent release into the culture supernatant, thus directly contributing to the chemotactic environment during Mtb-infection (Fig 9B). In fact, in a transwell chemotaxis assay, confirmed, that the epithelial-driven differences in the mediator environment contributed to cellular influx towards the site of infection. The largest population in peripheral blood are polymorphonuclear (PMN) cells (S6 Fig), of which neutrophils are rapid responders during bacterial infections [26]. PMN migration towards cell free cell-culture supernatants, which was generated in the same ways as the supernatants for secretome analysis, was measured. Supernatants from infected epithelial-myeloid co-cultures attracted on average approximately 9-fold more PMNs than supernatants from infected THP-1 monocultures (Fig 9C–9F), suggesting that epithelial cells contribute crucially to the leukocyte recruitment to the site of infection.

## Discussion

The first step in the natural history of tuberculosis infection, the host-pathogen interaction in the human airways after Mtb inhalation, is likely shaped by the two dominant cell types in the lungs: epithelial cells and alveolar macrophages. While macrophages are well known to respond and interact with Mtb, airway epithelial cells are understudied. We present the first global transcriptomic profiling of primary human airway epithelial cells in response to Mtb and Mtb-driven inflammation and found that epithelial cells contribute distinctly to the host response against Mtb through antimicrobial effectors as well as by amplifying the chemotactic environment initiated by infected macrophages to shape the local immunological environment.

To interrogate the epithelium in an *in vitro* setting which resembles early immune interactions in the human respiratory tract more physiologically, we established a co-culture model to allow contact-independent communication between PBECs and infected myeloid cells in real time. We used THP-1 cells to model myeloid responses in the co-culture system developed here and confirmed that the epithelial expression patterns are the same in response to Mtb-infected alveolar macrophages or THP-1 cells.

Through biological over-representation analysis of the epithelial transcriptome, we have uncovered the paracrine activation of epithelial cells by type I IFNs and IL1β. The interplay of type I IFN and IL1 has recently been elegantly delineated within infected macrophages and has been proposed as a point of therapeutic intervention during active tuberculosis [27]. While the cross-regulation of IL1 and type I IFN signalling in the Mtb-infected macrophage is an important well-documented rapidly-evolving area, our findings revealed for the first time that these pathways also act on the epithelial lining. In contrast to the induction patterns of IL1 and type I IFN within macrophages [20] and their respective cross-regulation, the paracrine epithelial activation induced by these two mediators occurred independently of each other with abrogation of either signalling pathway leaving gene expression of the opposite mediator untouched.

The activation of epithelial cells through Mtb-driven myeloid-derived type I IFNs has not previously been reported. Despite the association of type I IFNs with neutrophil-driven disease severity of tuberculosis in humans [28] and mice [29], the effect of type I IFNs on the outcome of Mtb-infection early after exposure is yet to be determined. Early myeloid-driven activation of IFN-signalling may be beneficial for the host as evidenced by the finding that IFN I and IFNγ are both required for optimal immune cell recruitment during the initial pre-adaptive phase of Mtb-infection in a mouse model [30]. Our findings further support this concept and showed that type I IFN signalling is required to induce epithelial transcription of the chemotactic factor *CXCL10* during Mtb-driven inflammation in humans. This activation, together with IL1β-mediated chemokine expression, amplifies cellular influx initiated by infected myeloid cells.

We observed that PBECs significantly enhance the neutrophil influx induced by infected myeloid cells and suggest that they may support rapid recruitment to the initial site of infection *in vivo*. Neutrophils are important early antimicrobial effectors with poor specificity. While they can cause substantial tissue damage and inflammation, their antimicrobial properties are crucial to control and prevent recurrent infections in humans [31]. Tissue neutrophilia is associated with pathogenesis during active TB; however, influx of neutrophils early after infection has been suggested to be beneficial for host control of the pathogen. Absence of neutrophils during early mycobacterial infection results in increased bacterial burden in mice and zebrafish [32,33] and growth control of mycobacteria by whole blood *in vitro* is strongly dependent on neutrophils [34] which may be an important function at the site of infection.

Besides the release of chemokines, myeloid-derived IL1β mediated the antimicrobial responses elicited in PBECs, including induction of *DEFB4* expression, in accordance with previously reported findings [9]. We further confirmed that DEFB4 was a potent direct anti-mycobacterial effector. While β-defensins are known to kill mycobacteria [17], IL1β-induced S100-family members have not previously been considered to be active against Mtb. Whilst both *S100A7A* (encoding koebnerisin) and *S100A7* (encoding psoriasin) are antibacterial effectors in the skin, neither were known to have antimycobacterial activity prior to our current discovery that psoriasin limited Mtb growth in liquid culture.

IL1β which mediated AMP-expression, is increased in the airways during active TB [35]; and may already be elevated early after Mtb-infection of alveolar macrophages before the onset of detectable lung pathology. This would then result in the activation of epithelial antimicrobial responses at the earliest stages of the natural history of tuberculosis in the human airways. Interestingly, IL1β -dependent epithelial AMP-expression was further enhanced by TNF in PBECs during co-culture with infected myeloid cells. While augmentation of TNF signalling by IL1β has been described in macrophages before [36], we believe that this is the first time that IL1β activation was shown to be enhanced by TNF in Mtb-infection. Epithelial antimicrobial peptides during infection may contribute to host defense against free extracellular mycobacteria thus preventing or diminishing cell to cell spread in early infection. They may also be a promising therapeutic target, since their activity is not affected by multidrug resistance of Mtb. Additionally, the identification of epithelial-specific AMP-expression with anti-mycobacterial activity may complement myeloid responses to Mtb in the airways, since monocytes and macrophages do not express high levels of the AMPs identified here [37,38].

Prior studies of macrophage-driven activation of epithelial cells focussed solely on specific pathways [9,11], involving matrix metalloproteinases (MMPs) or *DEFB4*. Our data provide the first global perspective on the epithelial response to Mtb infection, and reveal important new targets in the epithelial response to Mtb-infection while simultaneously confirming the previously published findings. In transwell co-culture, a large proportion of the expression signature (112 genes) was associated with the innate immune response, including type I IFN and cytokine-mediated signalling. *IL36G*, which is an emerging IL1-family member, was dependent on myeloid-derived IL1 β. It has recently been described to have a host-protective role during Mtb-infection [39] and its epithelial release may support macrophage function further. As part of the type I interferon-inducible mediators, the known antiviral factor *ISG15* was upregulated and its secretion during mycobacterial infection is involved in the appropriate induction of IFNγ in humans [40]. These are only two of several genes uncovered by the co-culture model we have established, which provide interesting targets for future investigations, but where further analysis into their function was beyond the scope of this study.

In contrast to their multifaceted responses to myeloid Mtb-infection, PBECs were surprisingly inert to direct stimulation with Mtb. This was not due to a general non-responsiveness to PRR-stimulation, as PBECs mount a substantial IL8 response towards synthetic TLR-agonists and in response to *Streptococcus pneumoniae* (Reuschl et al, *manuscript in preparation*). The global assessment of the transcriptomic response to direct stimulation with live Mtb revealed that no genes were significantly upregulated after 24h and this was complemented by poor invasion and adherence of Mtb to epithelial cells. This finding is consistent with reports of poor invasion of Mtb into primary tracheal epithelial cells in comparison to dendritic cells [41]. Interestingly, two days after *intra tracheal* Mtb-infection of mice, which results in locally very high density of Mtb bacilli, the majority of bacilli are found in macrophages and only approximately 10% of intracellular Mtb can be detected in epithelial cells [13]. Epithelial inertia to Mtb and the lack of uptake in humans may thus ensure the uptake of Mtb by local professional phagocytes, the preferred target host cell of Mtb after inhalation in the human lungs. Our co-culture model did not include endothelial cells and it remains to be seen whether interaction with an endothelial layer would augment the epithelial response to Mtb. Our findings contrast with previous studies, which described epithelial susceptibility to infection and upregulation of chemokines in A549 cells within 24h [10,42–44]. This most likely reflects differences between primary cells and cell lines, as A549 cells are adenocarcinoma-derived and may not truly mimic the responses of healthy human respiratory epithelium [45]. Through the recovery of PBECs from several donors, we have overcome the limitations of using a single cell line and reflected true biological variation in humans.

*In vivo*, the airway epithelium is at an air-liquid-interface, polarised and differentiated. However, in our model, the epithelial cells were undifferentiated and in submerged culture which allowed for comparison with the existing literature. Mediator release of undifferentiated epithelium to viral infection or air pollution particles surpasses the release by differentiated epithelium [46,47] suggesting that the inert phenotype of PBECs in response to Mtb is not an underestimate of their *in vivo* response and is moreover consistent with the lack of epithelial infection observed in mice *in vivo* [13].

We expanded and studied human primary cells *in vitro*, as opposed to using respiratory epithelial cell lines or mouse models. A limitation of our study is the focus on primary bronchial epithelial cells which, unlike alveolar epithelial cells, can be obtained by bronchoscopy. The recovery of pure alveolar epithelial cells requires samples from resections of dissected lungs or post-mortem cadaveric explants which may not reflect the responsiveness of healthy epithelium, and their phenotype is difficult to maintain *in vitro* [48]. Primary alveolar epithelial cells should be used to address whether our findings extend to human alveolar epithelial cells. This would be of interest as differences in the responsiveness to pro-inflammatory mediators between upper and lower airway epithelial cells have been previously described [49,50]. Myeloid-epithelial cross-talk was independent of cell-cell contact, providing evidence that the bronchial epithelium is able to respond to Mtb-infection upon activation by secreted mediators which diffuse from the site of infection throughout the airspaces. In the future, it would be important to assess the mechanisms identified in our study in PBECs derived from patients with active pulmonary tuberculosis, where epithelial responsiveness may be altered. Most of the epithelial cells within the human airways will not be in direct contact with the small number of infected alveolar macrophages. The majority of the initial epithelial response would therefore be expected to be driven by soluble mediators. Our experimental rationale therefore focussed on contact-independent interactions between infected myeloid and primary epithelial cells. Based on our findings, future work should also include direct interactions between the myeloid and epithelial compartment during infection to assess how cell-cell contact modulates the response to Mtb.

Our findings shed light on the contribution of the respiratory epithelial lining to the response against Mtb as well as the complexity of the immune response in the airways early after infection. It is known from challenge studies with rhinovirus in humans and *ex vivo* infection of bronchial tissue that only a limited number of epithelial cells are actively infected [51,52], yet potent immune activation occurs throughout the epithelial lining. Given that infection with Mtb requires only very few aerosolised mycobacteria [53,54], amplification of the macrophage-response to infection by uninfected surrounding epithelium may very well be decisive for the outcome of infection. Appropriate animal models of Mtb-infection should be employed to assess the reported epithelial responses *in vivo* during early infection.

In summary, we show that PBECs are inert to direct early Mtb-infection, but potent responders to infected alveolar macrophages. PBECs contributed to host defense as part of an Mtb-activated immune network through pathways, which are likely protective early after infection, by creating an extracellular antimicrobial milieu and promoting early neutrophil influx. Through these cell-specific differential responses to myeloid-derived cytokines, airway epithelial cells likely fulfil a non-redundant role in the human pulmonary host response to Mtb as vital players with the potential to shape the human innate immune response to infection and influence the outcome of exposure to Mtb.

## Methods

### Recruitment and ethics statement

PBECs and AMΦs were collected through bronchial brushings and bronchoalveolar lavage from the airways of healthy volunteers during bronchoscopic procedure at St. Mary’s Hospital, London, United Kingdom. All volunteers were adults and recruited through advertisements or from Contact and TB clinics at Imperial College NHS Trust (St. Mary’s Hospital), London North West Healthcare NHS Trust (Ealing Hospital, Northwick Park Hospital) and Barts Health NHS Trust (Newham Chest Clinic). All samples were collected in accordance with the Human Tissue Act 2004 and written informed consent was obtained from all participants (National Research Ethics Service approval reference 07/H0712/85+5).

### Cell culture

Bronchial epithelium was recovered from the right lower lobe of healthy volunteers through cytological brushes (Olympus Keymed, Southend-on-Sea, UK). PBECs were cultured from up to three brushes per volunteer and expanded in supplemented bronchial epithelial growth medium (BEGM) (Lonza, Walkersville, USA) as previously described [55]. To prevent the outgrowth of recovered contaminating bacteria or fungi, the growth medium was supplemented with gentamycin and amphothericin-B as per the manufacturer’s instruction. All cultures showed the characteristic cobblestone appearance of bronchial epithelial cells. PBECs were used in experiments after two passages. Cells were seeded at 8x10^4^/ml in collagen/fibrinogen-coated tissue culture plates and grown to confluence. BEGM was then changed to bronchial epithelial basal medium (BEBM) over night before experimentation. To identify cell subsets in the epithelial lining, *ex vivo* differential cell counts of one separate brush was obtained. Sample sizes given in figure legends refer to biological replicates from independent donors.

Alveolar macrophages (AMΦ) were recovered from bronchoalveolar lavage and isolated through overnight adherence to tissue culture plastic. AMΦ were cultured in RPMI with 10% human serum and 50μg/ml gentamycin. For infection experiments, medium was changed to medium with 5% human serum or BEBM. AMΦ were used at 10^6^/ml for experiments.

Peripheral blood leukocytes (PBL) were isolated from heparinised whole blood. Red blood cells were removed trough addition of 10 fold excess RBC lysis buffer (Biolegend, San Diego, USA) for 10 min.

THP-1 cells were obtained from American Type Culture Collection (ATCC) and grown RPMI with 10% FBS, 50000 U penicillin, 50 mg streptomycin (Sigma-Aldrich, Poole, UK) and 0.05 mM β-mercaptoethanol. For macrophage experiments, THP-1 cells were differentiated with 50nM phorbol 12-myristate 13-acetate (PMA) for 24h, seeded in tissue culture plates and rested overnight in complete medium without PMA. THP-1 cells were used at 10^6^/ml for experiments. Before infection experiments, culture medium was replaced with infection medium containing 5% human serum or BEBM.

### Mycobacterial culture

Mtb H37Rv, *M*. *bovis* BCG (SSI) or the clinical isolates Mtb CH [56] and Mtb NPH4216 [57] were cultured in 7H9 Middlebrook medium supplemented with 10% OADC, 0.5% glycerol, 0.05% Tween-80 and 10 μ g/ml amphotericin. Cultures were harvested in mid-log phase and frozen down in 15% glycerol. To standardise infection doses, cells were infected from frozen stocks and multiplicity of infections determined based on the mycobacterial counts of the stocks. For determination of colony forming units (CFU), mycobacteria were grown on 7H10 agar plates supplemented with 10% OADC, 0.5% glycerol and 0.05% Tween-80 and cultured for 3–4 weeks at 37°C. Antimycobacterial effects of selected peptides were performed in 96 well plates. hBD2 (PeproTech, London, UK), S100A7/Psoriasin (a kind gift from Prof. Joachim Grötzinger [23]) and controls were diluted in fresh 7H9 and Mtb bacilli harvested in mid-log phase was added. Plates were incubated shaking for up to 7 days at 36°C. Culture growth was monitored by measuring optical density at 595nm. OD-readings from 7H9-only controls were subtracted from all culture conditions before the data was analysed. On day 7, cultures were plated on 7H10 for CFU enumeration.

### Mtb-infection experiments

For infections, Mtb bacilli were diluted in cell culture medium, washed by centrifugation and taken up in cell culture medium for infection experiments. Mtb was sonicated for 40 seconds in an ultrasonic waterbath (Grant) to disperse clumps before inoculation of cell culture. For Mtb-uptake and adherence to THP-1 MΦ or PBECs. Mtb bacilli were added onto cells for 24h. Infection was determined by cell lysis with 0.1% Triton-X/PBS-Tween80. Lysates were serially diluted in PBS-Tween80 and plated on 7H10 agar. Association of Mtb with PBECs was visualised for representative experiments by Kinyoun stain with a Tb-color kit (Merck, Darmstadt, Germany) and images were acquired with an Axio Scope. A1 microscope (Zeiss, Rugby, UK). For some experiments, 200 μg/ml amikacin (Sigma) was added for 2h before cell lysis. To measure mediator release or gene expression, myeloid cells or PBECs were infected with Mtb at the indicated MOI and supernatants or RNA harvested at the indicated time.

For transwell experiments, PBECs were seeded into tissue culture plates then 0.4 μm cell culture inserts (Millipore) were placed on top with THP-1 cells added to them. Mtb was added to either compartment as desired (MOI5 over THP-1). For blocking and neutralisation of cytokine signalling, 20 μg/ml of antibodies (αIFNα/ β R2 (MMHAR-2, R&D Systems), α IFNγ (K3.53, R&D Systems), α IL1β (2805, R&D Systems), α TNF (1825, R&D Systems), mIgG1 (11711, R&D), mIgG2A (20102, R&D Systems) were added to the bottom well 45 min before transwell inserts and THP-1 cells were added. Culture supernatants were harvested from the tissue culture wells after inserts were removed.

### Supernatant transfer experiments

PBECs were exposed to soluble mediators released from uninfected or infected AMФ. Culture supernatants from AMФ or cell-free incubation controls ± Mtb bacilli were harvested after 24h and 0.22 μm sterile filtered. Supernatants were diluted 1:10 in fresh BEBM, added to PBECs and total RNA was harvested after 24h.

### Microarrays and RT-PCR

RNA was extracted with the TRIzol Plus RNA Purification Kit (Ambion) and treated with DNAse I (Thermo, Epsom, UK) to remove residual genomic DNA. For RT-PCR, RNA quality and quantity was measured by Nanodrop. RNA was converted to cDNA using Maxima reverse transcriptase and random hexamers. Solaris Gene Expression and Taqman Expression assays were used to determine gene expression levels by RT-PCR. RT-PCR reactions were performed in duplicate with 12.5 ng of cDNA. Expression data were analysed with Biogazelle qbase+ and normalised to *ACTB* and *PGK* according to [58]. Solaris Gene Expression assays: *ACTB* (AX-003451), *CXCL10* (AX-007871), *DEFB4A* (AX-012997), *IFIT1* (AX-019616), *IFNB1* (AX-019656), *IL36G* (AX-007959), *IL8* (AX-004756), *PGK* (AX-006767), *S100A7A* (AX-027145). Taqman Expression assays: *S100A7* (Hs01923188_u1).

For microarrays, RNA quality was assessed by Bioanalyzer (Agilent, Stockport, UK) (RIN > 9 for all samples) and RNA was quantified by Qubit (Thermo). 50-100ng total RNA was prepared for GeneChip Human Transcriptome Array 2.0 with the GeneChip WT PLUS Reagent Kit (Affymetrix, High Wycombe, UK). Samples were hybridized and scanned at the MRC Genomics Laboratory (Hammersmith Campus, Imperial College London).

### Soluble mediators

Soluble mediators were measured in cell culture supernatants after double-filtration through 0.22 μm centrifuge spin filters. Human IL1β, IFNγ and IFNλ were measured by DuoSet ELISAs (R&D Systems). IFNβ was measured by VeriKine Human IFN Beta ELISA kit (pbl interferon source).

### Secretome analysis

The secretome of Mtb-infected THP-1 cells and Mtb-infected PBEC-THP-1 co-cultures was measured by liquid chromatography—mass spectrometry (LC-MS). Six paired cell culture supernatants were analysed using two independent PBEC cultures. Cell culture supernatants were harvested at 48h and sterile filtered twice through 0.22 μm centrifuge filters. 500 μl of culture supernatants were buffer exchanged twice with 450 μl of 2 M urea, 100 mM Tris-HCL (pH 8.0) using a 3 kDa nominal molecular weight limit cut-off spin filter (Millipore). A volume of 80 μl of sample was reduced with 5 mM DTT (Sigma) for 30 min at 60°C and alkylated with 10 mM of iodoacetamide (Sigma) in the dark at room temperature for 30 min. 1 μg Trypsin/Lys-C mix (Promega) was added for 18 h at 37°C. Samples were adjusted to 1% (v/v) trifluoroacetic acid (TFA) (Sigma)and peptide digests were purified using the C18 STop And Go Extraction (STAGE) tips [PMID: 16602707] and resuspended in 2% (w/v) acetonitrile, 0.1% (v/v) fomic acid for LC-MS. Samples were analysed using an EASY-nLC 1000 Liquid chromatography system coupled to a Q-Exactive mass spectrometer. The separation column and emitter was an EASY-Spray column, 50 cm x 75 μm ID, PepMap C18, 2 μm particles, 100 Å pore size. Buffer A was 2% acetonitrile, 0.1% formic acid and buffer B 100% (v/v) acetonitrile, 0.1% (v/v) formic acid. A gradient from 5% to 40% acetonitrile over 120 min was used to elute peptides for ionization by electrospray ionisation (ESI) and data dependent MS/MS acquisition consisting of 1 full MS1 (R = 70K) scan acquisition from 350-1500m/z, and 10 HCD type MS2 scans (R = 15K). MS/MS charge targets were limited to 1E6 and iosolation window set to 2.0 m/z, monoisotopic precursor selection, charge state screening and dynamic exclusion were enabled, charge states of +1, >4 and unassigned charge states were not subjected to MS2 fragmentation. Raw mass spectra were identified and quantified using Maxquant 1.5.15 using a 1% peptide and protein FDR. Searches were conducted against the Uniprot SwissProt database downloaded on 06/06/2014. The database was supplemented with common contaminant proteins introduced during proteomic experiments. Searches were specified as tryptic with 1 missed cleavage, 7 ppm precursor ion mass tolerance, 0.05 Da fragment ion mass tolerance, fixed modifications of carbamidomethylation (C), and variable modification of oxidation (M), acetylation (N-term, Protein). Protein Label free quantitation (LFQ) intensity measured in medium alone was subtracted from all paired samples, and LFQ values were log2 transformed. To identify both qualitative and quantitative effects of stimulation on extracellular all missing values were replaced assuming a normal distribution (width 0.3, and a down shift of 1.8) for only control/unstimulated samples as described in [59].

### Western blot

For protein lysates PBECs were treated with RIPA buffer (Pierce) supplemented with 2x Halt Protease and Phosphatase Inhibitor Cocktail (Pierce) and 250 U Benzonase (Sigma) for 15 min on ice. Lysates were centrifuged at 14000 rpm for 15 min at 4°C. Total protein concentration was measured by BCA protein assay (Pierce). 12 μg of protein lysates lysates were mixed with Laemmli buffer (BioRad, Hertfordshire, UK) and 25 mM DTT and boiled for 7 min. Proteins separated on 4%-20% Mini PROTEAN gels (BioRad) and transferred onto nitrocellulose membranes by iBlot (Invitrogen). Membranes were blocked in 5% milk/TBST for 1h and probed with primary antibodies (αbeta-Actin (D6A8, New England Biolabs), αPhospho-Stat1 (Tyr701) (D4A7, New England Biolabs), αStat1 (#9172, New England Biolabs)) at 1:1000 in 5% BSA/TBST over night at 4°C. HRP-linked IgG (New England Biolabs) (1:2000) in 5% BSA/TBST was added the next day for 1h. Membranes were developed with ECL Western blotting substrate (Pierce) and imaged with FUSION FX7 SPECTRA (Vilber).

### PBL phenotyping by flow cytometry

PBLs were isolated and resuspended in PBS with 0.5% BSA and 2mM EDTA (FACS buffer). Before staining, 10% human serum in FACS buffer was added to cells for 20 min to block Fc receptors. For surface staining, PBLs were incubated with α CD14-Brillian Violet 421 (M5E2, Biolegend), α CD15-Brillian Violet 605 (SSEA-1, Biolegend), α CD3-PE-CF594 (UCHT1, BD) and α CD66b-PerCP/Cy5.5 (G10F5, Biolegend). Cells were treated with Cytofix Fixation buffer, resuspendend in FACS buffer and left overnight before flow cytometric acquisition using either a BD LSR II or BD Fortessa. Anti-Mouse Ig compensation beads (BD) were used to determine compensation parameters.

### PBL transwell migration assay

Cell free culture supernatants were generated through direct co-culture of Mtb-infected THP-1 cells in the presence of absence of PBECs for 48h. BEBM was used as a background control for unspecific migration. Conditioned or control medium was added to the bottom well of a tissue culture plate and 0.5 μm transwell inserts (Corning) placed on top. The plates were placed in a humified 37°C CO_2_ incubator for 30 min to equilibrate. 2.5x10^5^ PBLs were added to the transwell insert and the plate was placed back into the incubator for cell migration. After 3h, 2mM EDTA was added to the bottom compartment to dislodge cells from the transwell membrane. Cells were collected from the insert and bottom compartment, fixed and resuspended in equal volumes of FACS buffer. Cells in each sample were enumerated through acquisition for 120 s using a BD LSR II flow cytometer.

### Statistical analysis

Microarray data was normalised by Robust Multi-Array Average (RMA) using Partek Genomic Suite 6. All genes annotated by NCBI Reference Sequence Database (RefSeq), NIH GenBank or Ensembl were taken forward for statistical analysis. One-Way ANOVA on microarray data was performed with PGS. For SAM analysis of expression and secretome data with TIGR MultiExperiment Viewer 256 or 64 permutations were used, respectively. Pathway analysis was performed with InnateDB for over-representation analysis [60]. Transcription factor binding site enrichment on differentially expressed genes was performed using oPossum [61].

Flow cytometry data was analysed using FlowJo v10. Statistical analysis of all other experiments was performed with GraphPad Prism 6 and is indicated where appropriate.

## Supporting information

S1 Fig Association of Mtb with PBECs visualised by Kinyoun stain. PBECs were infected with Mtb H37Rv (MOI50) for 24h. The association of Mtb with PBECs was confirmed microscopically via Kinyoun stain after 24h of infection at MOI 50. Shown are representative images from two donors at a 200x magnification. Arrowheads indicate Mtb.
(TIF)

S2 Fig PBEC gene expression in response to AMФ in transwell co-culture. AMФ from two healthy donors were co-cultured in the transwell model with PBECs and infected with Mtb H37Rv (MOI5) as indicated. Gene expression was measured by RT-PCR.
(TIF)

S3 Fig Interferon-release and gene expression in response to infection, transwell co-culture and recombinant cytokines. A)PBECs were stimulated in the transwell co-culture system as indicated and *IFNB* expression measured after 24h. Fold change was calculated over unstimulated cells. Boxplots show median and range (n = 5). No significant differences were detected.B)IFNγ and IFNλ release by THP-1 cells after 24h of Mtb-infection (MOI5) was measured by ELISA in two independent experiments. Mean ± SD. n.d., not detected; φ, extrapolated values below the detection limit; horizontal lines indicate the detection limits of the assays.C)PBECs were stimulated with 1 ng/ml IL1β or IFNβ for 24h and gene expression was measured by RT-PCR (n = 3). Mean ± SD are shown.D)PBECs were co-cultured with Mtb-infected THP-1 cells in the presence of 20 μg/ml αL1β or IgG_1_ (isotype control) as indicated. After 24h, gene expression was measured by RT-PCR. Expression is shown as fold change over unstimulated (n = 6). Boxplots show median and range.E)PBECs were co-cultured with Mtb-infected THP-1 cells in the presence of 20 μg/ml αIFNAR2 or IgG_2_ (isotype control). After 24h, gene expression was measured by RT-PCR and is shown as fold change over unstimulated (n = 3). Median is shown.
Friedman test with Dunn’s post-test was used to compare groups against isotype control. n.s., not significant; *, p<0.05.
(TIF)

S4 Fig Effects of TNF and IFNψ on PBEC-myeloid co-culture. (A)PBECs were co-cultured with Mtb-infected (MOI5) THP-1 cells with αTNF or IgG_1_ for 24h. *DEFB4* expression was measured by RT-PCR and is shown as fold change over unstimulated PBECs (n = 3).(B)IL1β release was measured in the co-culture supernatants of (A) by ELISA.(C)THP-1 MΦs were infected with Mtb (MOI5) in the presence of αTNF or IgG_1_ and IL1β release measured after 24h. Cytokine levels are shown as % of IL1 β release during infection in the presence of IgG_1_ (n = 5).(D)PBECs were exposed to Mtb-infected THP-1 cells (MOI5) in co-culture in the presence of αIFNγ or IgG_2a_. After 24h, *CXCL10* expression was measured by RT-PCR and is shown as fold change over unstimulated PBECs.
Mean ± SD are shown. (A, B and D) Wilcoxon signed rank test was used to compare groups; (C) was compared by repeat-measure ANOVA with Holm-Sidak's multiple comparisons test. **, p<0.01 or exact p-values are given.
(TIF)

S5 Fig Antimycobacterial effects of hBD2 and expression of *S100A7* in PBECs during transwell co-cultures. (A)Clinical isolates Mtb NPH4216 and Mtb CH were incubated with 5 μg/ml recombinant hBD2 or vehicle control as described in Fig 8. Colony forming units (CFU) were determined at day 7. Effects of hBD2 was compared with vehicle control by Student t-test. Mean ± SD of triplicate measurements are shown. * p<0.05; ** p<0.01(B)In the transwell model, PBECs were exposed to THP-1 cells or Mtb H37Rv (MOI5 over THP-1) for 24h as indicated. *S100A7* expression in PBECs was measured by RT-PCR and is shown as fold change over unstimulated PBECs (n = 5).(C)PBECs were co-cultured with infected or uninfected THP-1 cells in the presence of αL1β or IgG_1_ as indicated. After 24h, *S100A7* expression was measured by RT-PCR and is shown as fold change over unstimulated PBECs (n = 5).
Friedman test with Dunn’s post-test was used to compare expression with unstimulated or respective isotype control. Boxplots show median and range. * p<0.05; ** p<0.01.
(TIF)

S6 Fig Gating strategy for PBL transwell migration experiments. PBLs were isolated from whole blood and stained for CD3, CD14, CD15 and CD66b. Shown are representative plots for the gating strategy from one of three donors. After gating for singlets, forward (FSC) and side (SSC) scatter were used to define PBL subsets. PMN, polymorphonuclear cells.
(TIF)

S1 Table Differentially expressed genes in PBECs exposed to Mtb-infected THP-1 cells in transwell co-culture. Significantly differentially expressed genes at a q-value < 5% were determined by Significance Analysis of Microarrays.
(XLSX)

S2 Table Secretome of Mtb-infected THP-1 monocultures and co-cultures with PBECs. Significantly differentially secreted proteins identified in cell-free culture supernatants of Mtb-infected THP-1 cells co-cultured with PBECs compared to infected THP-1 monoculture at a q-value < 5% (determined by SAM).
(XLSX)

## References

[1] Morrison J, Pai M, Hopewell PC. Tuberculosis and latent tuberculosis infection in close contacts of people with pulmonary tuberculosis in low-income and middle-income countries: a systematic review and meta-analysis. Lancet Infect Dis. 2008;8: 359–68. doi: 10.1016/S1473-3099(08)70071-9 18450516 10.1016/S1473-3099(08)70071-9

[2] Cobat A, Gallant CJ, Simkin L, Black GF, Stanley K, Hughes J, et al Two loci control tuberculin skin test reactivity in an area hyperendemic for tuberculosis. J Exp Med. 2009;206: 2583–91. doi: 10.1084/jem.20090892 19901083 10.1084/jem.20090892 PMC2806605

[3] Verrall AJ, Netea MG, Alisjahbana B, Hill PC, van Crevel R. Early clearance of Mycobacterium tuberculosis: a new frontier in prevention. Immunology. 2014;141: 506–513. doi: 10.1111/imm.12223 24754048 10.1111/imm.12223 PMC3956425

[4] Thurlbeck WM. Internal surface area and other measurements in emphysema. Thorax. 1967;22: 483–96. 5624577 10.1136/thx.22.6.483 PMC471691

[5] Whitsett JA, Alenghat T. Respiratory epithelial cells orchestrate pulmonary innate immunity. Nat Immunol. Nature Publishing Group; 2014;16: 27–35. doi: 10.1038/ni.3045 25521682 10.1038/ni.3045 PMC4318521

[6] Bermudez L, Goodman J. Mycobacterium tuberculosis invades and replicates within type II alveolar cells. Infect Immun. 1996;64: 1400–1406. 8606107 10.1128/iai.64.4.1400-1406.1996 PMC173932

[7] Nouailles G, Dorhoi A, Koch M, Zerrahn J, Weiner J, Faé KC, et al CXCL5-secreting pulmonary epithelial cells drive destructive neutrophilic inflammation in tuberculosis. J Clin Invest. 2014;124: 1268–1282. doi: 10.1172/JCI72030 24509076 10.1172/JCI72030 PMC3934185

[8] Chuquimia OD, Petursdottir DH, Rahman MJ, Hartl K, Singh M, Fernández C. The role of alveolar epithelial cells in initiating and shaping pulmonary immune responses: communication between innate and adaptive immune systems. PLoS One. 2012;7: e32125 doi: 10.1371/journal.pone.0032125 22393384 10.1371/journal.pone.0032125 PMC3290547

[9] Verway M, Bouttier M, Wang T-T, Carrier M, Calderon M, An B-S, et al Vitamin D Induces Interleukin-1β Expression: Paracrine Macrophage Epithelial Signaling Controls M. tuberculosis Infection. Sassetti CM, editor. PLoS Pathog. 2013;9: e1003407 doi: 10.1371/journal.ppat.1003407 23762029 10.1371/journal.ppat.1003407 PMC3675149

[10] Wickremasinghe MI, Thomas LH, Friedland JS. Pulmonary epithelial cells are a source of IL-8 in the response to Mycobacterium tuberculosis: essential role of IL-1 from infected monocytes in a NF-kappa B-dependent network. J Immunol. 1999;163: 3936–47. 10490995

[11] Elkington PT, Green J A, Emerson JE, Lopez-Pascua LD, Boyle JJ, O’Kane CM, et al Synergistic up-regulation of epithelial cell matrix metalloproteinase-9 secretion in tuberculosis. Am J Respir Cell Mol Biol. 2007;37: 431–7. doi: 10.1165/rcmb.2007-0011OC 17575075 10.1165/rcmb.2007-0011OC

[12] Arriaga AK, Orozco EH, Aguilar LD, Rook GAW, Hernández Pando R. Immunological and pathological comparative analysis between experimental latent tuberculous infection and progressive pulmonary tuberculosis. Clin Exp Immunol. 2002;128: 229–237. doi: 10.1046/j.1365-2249.2002.01832.x 11985512 10.1046/j.1365-2249.2002.01832.x PMC1906395

[13] Sato K, Tomioka H, Shimizu T, Gonda T, Ota F, Sano C. Type II alveolar cells play roles in macrophage-mediated host innate resistance to pulmonary mycobacterial infections by producing proinflammatory cytokines. J Infect Dis. 2002;185: 1139–47. doi: 10.1086/340040 11930324 10.1086/340040

[14] Hernández-Pando R, Jeyanathan M, Mengistu G, Aguilar D, Orozco H, Harboe M, et al Persistence of DNA from Mycobacterium tuberculosis in superficially normal lung tissue during latent infection. Lancet. 2000;356: 2133–8. 11191539 10.1016/s0140-6736(00)03493-0

[15] Hirsch CS, Ellner JJ, Russell DG, Rich EA. Complement receptor-mediated uptake and tumor necrosis factor-alpha-mediated growth inhibition of Mycobacterium tuberculosis by human alveolar macrophages. J Immunol. 1994;152: 743–53. 8283049

[16] Carranza C, Juárez E, Torres M, Ellner JJ, Sada E, Schwander SK. Mycobacterium tuberculosis growth control by lung macrophages and CD8 cells from patient contacts. Am J Respir Crit Care Med. 2006;173: 238–45. doi: 10.1164/rccm.200503-411OC 16210664 10.1164/rccm.200503-411OC PMC2662991

[17] Corrales-Garcia L, Ortiz E, Castañeda-Delgado J, Rivas-Santiago B, Corzo G. Bacterial expression and antibiotic activities of recombinant variants of human β-defensins on pathogenic bacteria and M. tuberculosis. Protein Expr Purif. Elsevier Inc.; 2013;89: 33–43. doi: 10.1016/j.pep.2013.02.007 23459290 10.1016/j.pep.2013.02.007

[18] Büchau AS, Hassan M, Kukova G, Lewerenz V, Kellermann S, Würthner JU, et al S100A15, an antimicrobial protein of the skin: regulation by E. coli through Toll-like receptor 4. J Invest Dermatol. 2007;127: 2596–604. doi: 10.1038/sj.jid.5700946 17625598 10.1038/sj.jid.5700946

[19] Coussens AK, Wilkinson RJ, Martineau AR. Phenylbutyrate Is Bacteriostatic against Mycobacterium tuberculosis and Regulates the Macrophage Response to Infection, Synergistically with 25-Hydroxy-Vitamin D3. PLoS Pathog. 2015;11: e1005007 doi: 10.1371/journal.ppat.1005007 26133770 10.1371/journal.ppat.1005007 PMC4489717

[20] Novikov A, Cardone M, Thompson R, Shenderov K, Kirschman KD, Mayer-Barber KD, et al Mycobacterium tuberculosis triggers host type I IFN signaling to regulate IL-1β production in human macrophages. J Immunol. 2011;187: 2540–7. doi: 10.4049/jimmunol.1100926 21784976 10.4049/jimmunol.1100926 PMC3159798

[21] Stanley S A., Johndrow JE, Manzanillo P, Cox JS. The Type I IFN Response to Infection with Mycobacterium tuberculosis Requires ESX-1-Mediated Secretion and Contributes to Pathogenesis. J Immunol. 2007;178: 3143–3152. doi: 10.4049/jimmunol.178.5.3143 17312162 10.4049/jimmunol.178.5.3143

[22] Moreira-Teixeira L, Sousa J, McNab FW, Torrado E, Cardoso F, Machado H, et al Type I IFN Inhibits Alternative Macrophage Activation during Mycobacterium tuberculosis Infection and Leads to Enhanced Protection in the Absence of IFN-γ Signaling. J Immunol. 2016;197: 4714–4726. doi: 10.4049/jimmunol.1600584 27849167 10.4049/jimmunol.1600584 PMC5133670

[23] Jansen S, Podschun R, Leib SL, Grötzinger J, Oestern S, Michalek M, et al Expression and function of psoriasin (S100A7) and koebnerisin (S100A15) in the brain. Infect Immun. 2013;81: 1788–1797. doi: 10.1128/IAI.01265-12 23478321 10.1128/IAI.01265-12 PMC3648009

[24] Algood HMS, Lin PL, Yankura D, Jones A, Chan J, Flynn JL. TNF influences chemokine expression of macrophages in vitro and that of CD11b+ cells in vivo during Mycobacterium tuberculosis infection. J Immunol. 2004;172: 6846–57. doi: 10.4049/jimmunol.172.11.6846 15153503 10.4049/jimmunol.172.11.6846

[25] Ferrero E, Biswas P, Vettoretto K, Ferrarini M, Uguccioni M, Piali L, et al Macrophages exposed to Mycobacterium tuberculosis release chemokines able to recruit selected leucocyte subpopulations: focus on gammadelta cells. Immunology. 2003;108: 365–74 doi: 10.1046/j.1365-2567.2003.01600.x 12603603 10.1046/j.1365-2567.2003.01600.x PMC1782907

[26] Brinkmann V, Reichard U, Goosmann C, Fauler B, Uhlemann Y, Weiss DS, et al Neutrophil extracellular traps kill bacteria. Science. 2004;303: 1532–1535. doi: 10.1126/science.1092385 15001782 10.1126/science.1092385

[27] Mayer-Barber KD, Andrade BB, Oland SD, Amaral EP, Barber DL, Gonzales J, et al Host-directed therapy of tuberculosis based on interleukin-1 and type I interferon crosstalk. Nature. Nature Publishing Group; 2014;511: 99–103. doi: 10.1038/nature13489 24990750 10.1038/nature13489 PMC4809146

[28] Berry MPR, Graham CM, McNab FW, Xu Z, Bloch S a a, Oni T, et al An interferon-inducible neutrophil-driven blood transcriptional signature in human tuberculosis. Nature. Nature Publishing Group; 2010;466: 973–7. doi: 10.1038/nature09247 20725040 10.1038/nature09247 PMC3492754

[29] Dorhoi A, Yeremeev V, Nouailles G, Weiner J, Jörg S, Heinemann E, et al Type I IFN signaling triggers immunopathology in tuberculosis-susceptible mice by modulating lung phagocyte dynamics. Eur J Immunol. 2014;44: 2380–2393. doi: 10.1002/eji.201344219 24782112 10.1002/eji.201344219 PMC4298793

[30] Desvignes L, Wolf AJ, Ernst JD. Dynamic roles of type I and type II IFNs in early infection with Mycobacterium tuberculosis. J Immunol. 2012;188: 6205–15. doi: 10.4049/jimmunol.1200255 22566567 10.4049/jimmunol.1200255 PMC3370955

[31] Bianchi M, Hakkim A, Brinkmann V, Siler U, Seger R a., Zychlinsky A, et al Restoration of NET formation by gene therapy in CGD controls aspergillosis. Blood. 2009;114: 2619–2622. doi: 10.1182/blood-2009-05-221606 19541821 10.1182/blood-2009-05-221606 PMC2756123

[32] Yang C-T, Cambier CJ, Davis JM, Hall CJ, Crosier PS, Ramakrishnan L. Neutrophils exert protection in the early tuberculous granuloma by oxidative killing of mycobacteria phagocytosed from infected macrophages. Cell Host Microbe. Elsevier Inc.; 2012;12: 301–12. doi: 10.1016/j.chom.2012.07.009 22980327 10.1016/j.chom.2012.07.009 PMC3638950

[33] Pedrosa J, Saunders BM, Appelberg R, Orme IM, Silva MT, Cooper a M. Neutrophils play a protective nonphagocytic role in systemic Mycobacterium tuberculosis infection of mice. Infect Immun. 2000;68: 577–83. doi: 10.1128/IAI.68.2.577-583.2000 10639420 10.1128/iai.68.2.577-583.2000 PMC97179

[34] Martineau AR, Newton SM, Wilkinson KA, Kampmann B, Hall BM, Nawroly N, et al Neutrophil-mediated innate immune resistance to mycobacteria. J Clin Invest. American Society for Clinical Investigation; 2007;117: 1988–94. doi: 10.1172/JCI31097 17607367 10.1172/JCI31097 PMC1904316

[35] Thillai M, Eberhardt C, Lewin AM, Potiphar L, Hingley-Wilson S, Sridhar S, et al Sarcoidosis and tuberculosis cytokine profiles: indistinguishable in bronchoalveolar lavage but different in blood. PLoS One. 2012;7: e38083 doi: 10.1371/journal.pone.0038083 22815689 10.1371/journal.pone.0038083 PMC3398021

[36] Jayaraman P, Sada-Ovalle I, Nishimura T, Anderson AC, Kuchroo VK, Remold HG, et al IL-1β promotes antimicrobial immunity in macrophages by regulating TNFR signaling and caspase-3 activation. J Immunol. 2013;190: 4196–204. doi: 10.4049/jimmunol.1202688 23487424 10.4049/jimmunol.1202688 PMC3622150

[37] Rivas-Santiago B, Schwander SK, Diamond G, Klein-patel ME, Ellner JJ, Sada E, et al Human β -Defensin 2 Is Expressed and Associated with Mycobacterium tuberculosis during Infection of Human alveolar epithelial cells. Infect Immun. 2005;73: 4505–4511. doi: 10.1128/IAI.73.8.4505-4511.2005 16040961 10.1128/IAI.73.8.4505-4511.2005 PMC1201238

[38] Lee KC, Eckert RL. S100A7 (Psoriasin)—mechanism of antibacterial action in wounds. J Invest Dermatol. 2007;127: 945–57. doi: 10.1038/sj.jid.5700663 17159909 10.1038/sj.jid.5700663

[39] Ahsan F, Moura-alves P, Guhlich-bornhof U, Klemm M, Kaufmann SHE, Maertzdorf J. Role of Interleukin 36 γ in Host Defense Against Tuberculosis. 2016; 1–11. doi: 10.1093/infdis/jiw152 27389350 10.1093/infdis/jiw152

[40] Bogunovic D, Byun M, Durfee LA, Abhyankar A, Sanal O, Mansouri D, et al Mycobacterial disease and impaired IFN-γ immunity in humans with inherited ISG15 deficiency. Science. 2012;337: 1684–8. doi: 10.1126/science.1224026 22859821 10.1126/science.1224026 PMC3507439

[41] Harriff MJ, Cansler ME, Toren KG, Canfield ET, Kwak S, Gold MC, et al Human lung epithelial cells contain Mycobacterium tuberculosis in a late endosomal vacuole and are efficiently recognized by CD8^+^ T cells. PLoS One. 2014;9: e97515 doi: 10.1371/journal.pone.0097515 24828674 10.1371/journal.pone.0097515 PMC4020835

[42] Lee H-M, Shin D-M, Jo E-K. Mycobacterium tuberculosis Induces the Production of Tumor Necrosis Factor-α, Interleukin-6, and CXCL8 in Pulmonary Epithelial Cells Through Reactive Oxygen Species-dependent Mitogen-activated Protein Kinase Activation. J Bacteriol Virol. 2009;39: 10 doi: 10.4167/jbv.2009.39.1.1

[43] Lin Y, Zhang M, Barnes PF. Chemokine production by a human alveolar epithelial cell line in response to Mycobacterium tuberculosis. Infect Immun. 1998;66: 1121–6. 9488404 10.1128/iai.66.3.1121-1126.1998 PMC108024

[44] Mehta PK, King CH, White EH, M JJ Jr, Quinn FD, Murtagh JJ, et al Comparison of in vitro models for the study of Mycobacterium tuberculosis invasion and intracellular replication. Infect Immun. 1996;64(7):2673 8698494 10.1128/iai.64.7.2673-2679.1996 PMC174125

[45] Giard DJ, Aaronson S A, Todaro GJ, Arnstein P, Kersey JH, Dosik H, et al In vitro cultivation of human tumors: establishment of cell lines derived from a series of solid tumors. J Natl Cancer Inst. 1973;51: 1417–1423. doi: 10.1093/jnci/51.5.1417 4357758 10.1093/jnci/51.5.1417

[46] Lopez-Souza N, Favoreto S, Wong H, Ward T, Yagi S, Schnurr D, et al In vitro susceptibility to rhinovirus infection is greater for bronchial than for nasal airway epithelial cells in human subjects. J Allergy Clin Immunol. Elsevier Ltd; 2009;123: 1384–90.e2. doi: 10.1016/j.jaci.2009.03.010 19428098 10.1016/j.jaci.2009.03.010 PMC2744461

[47] Ghio AJ, Dailey L a, Soukup JM, Stonehuerner J, Richards JH, Devlin RB. Growth of human bronchial epithelial cells at an air-liquid interface alters the response to particle exposure. Part Fibre Toxicol. Particle and Fibre Toxicology; 2013;10: 25 doi: 10.1186/1743-8977-10-25 23800224 10.1186/1743-8977-10-25 PMC3750262

[48] Mao P, Wu S, Li J, Fu W, He W, Liu X, et al Human alveolar epithelial type II cells in primary culture. Physiol Rep. 2015;3: 1–12. doi: 10.14814/phy2.12288 25677546 10.14814/phy2.12288 PMC4393197

[49] Sauty a, Dziejman M, Taha R a, Iarossi a S, Neote K, Garcia-Zepeda E a, et al The T cell-specific CXC chemokines IP-10, Mig, and I-TAC are expressed by activated human bronchial epithelial cells. J Immunol. 1999;162: 3549–58. 10092813

[50] Thomas LH, Wickremasinghe MIY, Friedland JS. IL-1 beta stimulates divergent upper and lower airway epithelial cell CCL5 secretion. Clin Immunol. 2007;122: 229–38. doi: 10.1016/j.clim.2006.10.004 17126080 10.1016/j.clim.2006.10.004

[51] Mosser AG, Brockman-Schneider R, Amineva S, Burchell L, Sedgwick JB, Busse WW, et al Similar frequency of rhinovirus-infectible cells in upper and lower airway epithelium. J Infect Dis. 2002;185: 734–743. doi: 10.1086/339339 11920291 10.1086/339339

[52] Arruda E, Boyle TR, Winther B, Pevear DC, Gwaltney JM, Hayden FG. Localization of Human Rhinovirus Replication in the Upper Respiratory Tract by In Situ Hybridization. J Infect Dis. 1995;171: 1329–1333. doi: 10.1093/infdis/171.5.1329 7751712 10.1093/infdis/171.5.1329

[53] Dean GS, Rhodes SG, Coad M, Whelan a O, Cockle PJ, Clifford DJ, et al Minimum effective dose of Mycobacterium bovis in cattle. InfectImmun. 2005;73: 6467–6471. 10.1128/IAI.73.10.6467-6471.2005 PMC1230957 16177318

[54] Riley RL. Aerial dissemination of pulmonary tuberculosis. Am Rev Tuberc. 1957;76: 931–41. 13488004 10.1164/artpd.1957.76.6.931

[55] Edwards MR, Regamey N, Vareille M, Kieninger E, Gupta A, Shoemark A, et al Impaired innate interferon induction in severe therapy resistant atopic asthmatic children. Mucosal Immunol. Nature Publishing Group; 2013;6: 797–806. doi: 10.1038/mi.2012.118 23212197 10.1038/mi.2012.118 PMC3684776

[56] Newton SM, Smith RJ, Wilkinson K a, Nicol MP, Garton NJ, Staples KJ, et al A deletion defining a common Asian lineage of Mycobacterium tuberculosis associates with immune subversion. Proc Natl Acad Sci U S A. 2006;103: 15594–15598. doi: 10.1073/pnas.0604283103 17028173 10.1073/pnas.0604283103 PMC1622867

[57] Anderson ST, Williams AJ, Brown JR, Newton SM, Simsova M, Nicol MP, et al Transmission of Mycobacterium tuberculosis undetected by tuberculin skin testing. Am J Respir Crit Care Med. 2006;173: 1038–1042. doi: 10.1164/rccm.200509-1526OC 16456140 10.1164/rccm.200509-1526OC

[58] Hellemans J, Mortier G, De Paepe A, Speleman F, Vandesompele J. qBase relative quantification framework and software for management and automated analysis of real-time quantitative PCR data. Genome Biol. 2007;8: R19 doi: 10.1186/gb-2007-8-2-r19 17291332 10.1186/gb-2007-8-2-r19 PMC1852402

[59] Meissner F, Scheltema RA, Mollenkopf H, Mann M. Direct Proteomic Quantification of the Secretome of Activated Immune Cells. 2013; 475–479. 10.1126/science.1232578 23620052

[60] Breuer K, Foroushani AK, Laird MR, Chen C, Sribnaia A, Lo R, et al InnateDB: Systems biology of innate immunity and beyond—Recent updates and continuing curation. Nucleic Acids Res. 2013;41: D1228–33. doi: 10.1093/nar/gks1147 23180781 10.1093/nar/gks1147 PMC3531080

[61] Ho Sui SJ, Mortimer JR, Arenillas DJ, Brumm J, Walsh CJ, Kennedy BP, et al oPOSSUM: Identification of over-represented transcription factor binding sites in co-expressed genes. Nucleic Acids Res. 2005;33: 3154–3164. doi: 10.1093/nar/gki624 15933209 10.1093/nar/gki624 PMC1142402

