# Supplementary figures and images for: Innate activation of human primary epithelial cells broadens the host response to Mycobacterium tuberculosis in the airways
Source: PLoS Pathog. 2017 Sep 1;13(9):e1006577. doi: 10.1371/journal.ppat.1006577 (PMC5605092; doi:10.1371/journal.ppat.1006577)

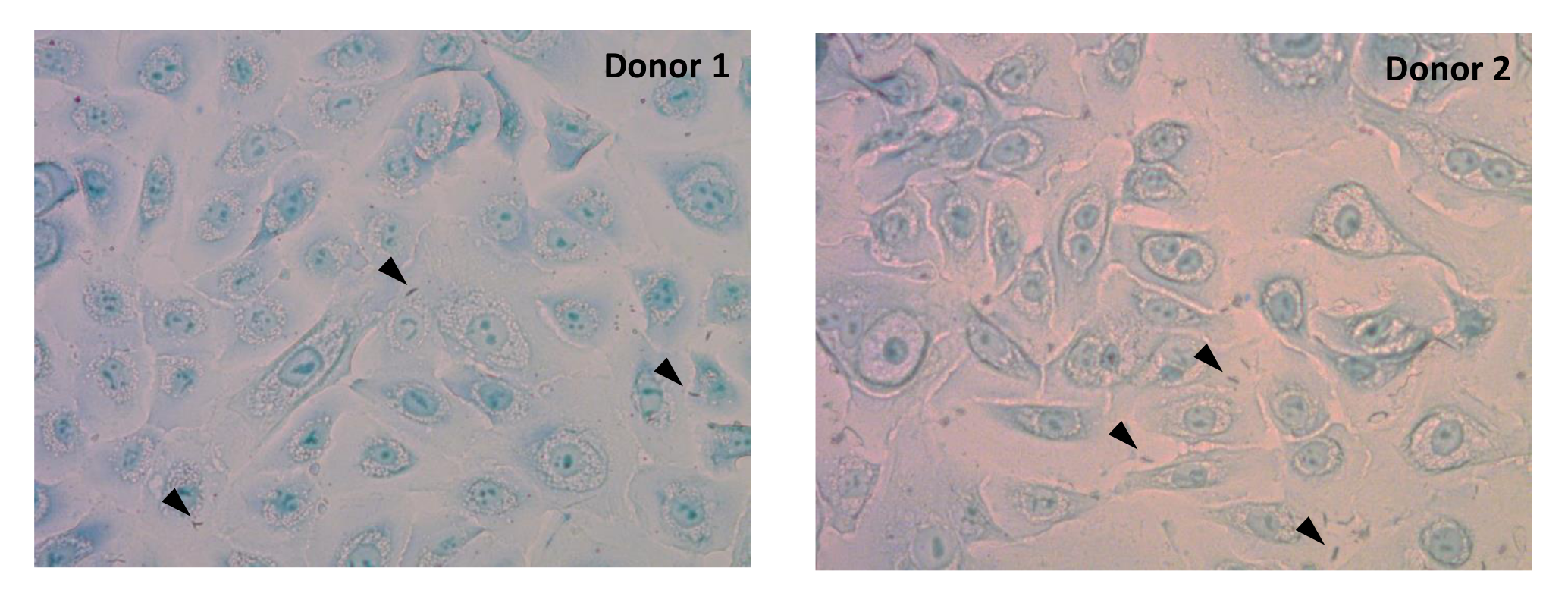

Supplement: S1 Fig — PBECs were infected with Mtb H37Rv (MOI50) for 24h. The association of Mtb with PBECs was confirmed microscopically via Kinyoun stain after 24h of infection at MOI 50. Shown are representative images from two donors at a 200x magnification. Arrowheads indicate Mtb. (TIF) [file ppat.1006577.s001.tif]

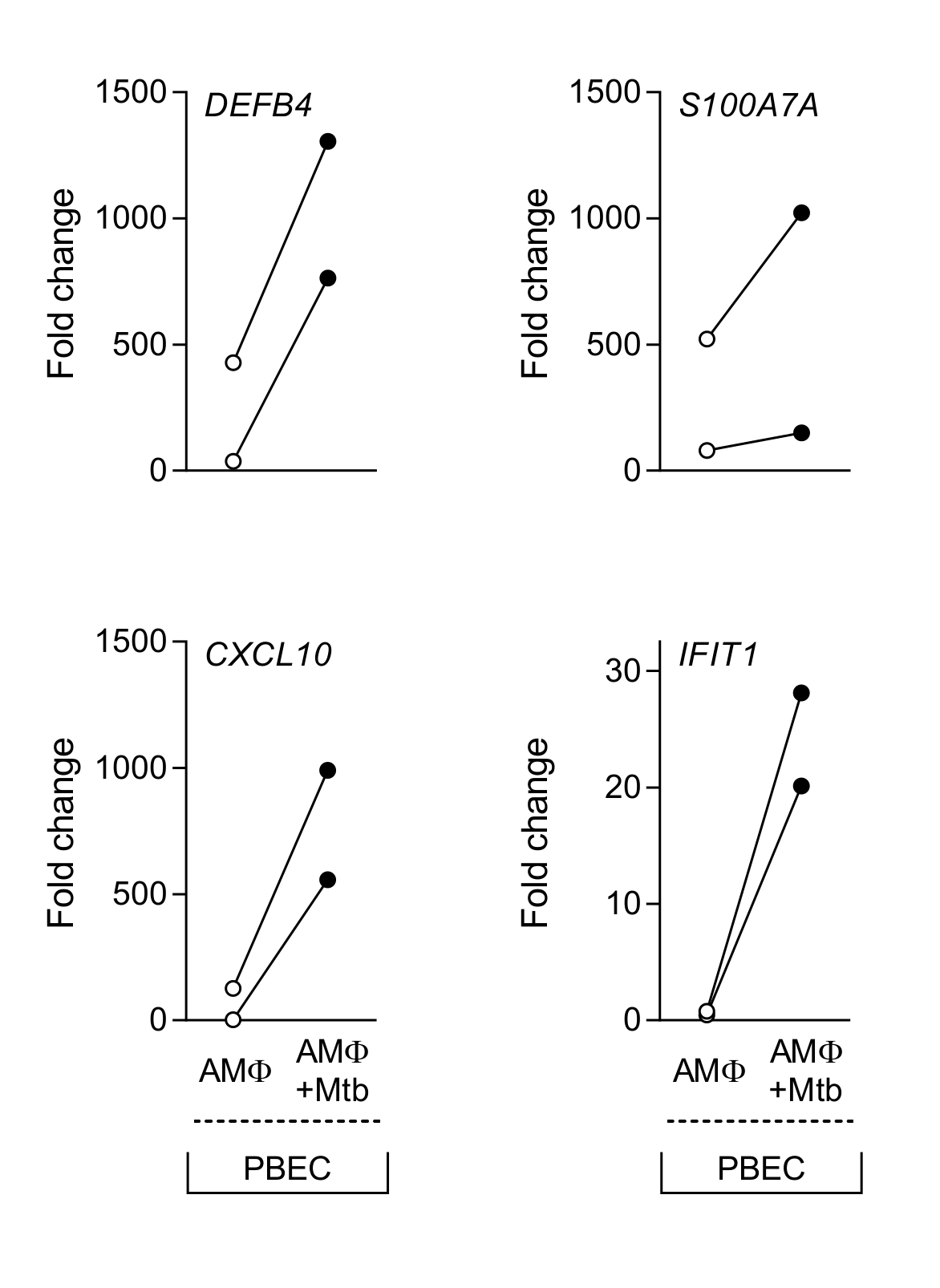

Supplement: S2 Fig — AMФ from two healthy donors were co-cultured in the transwell model with PBECs and infected with Mtb H37Rv (MOI5) as indicated. Gene expression was measured by RT-PCR. (TIF) [file ppat.1006577.s002.tif]

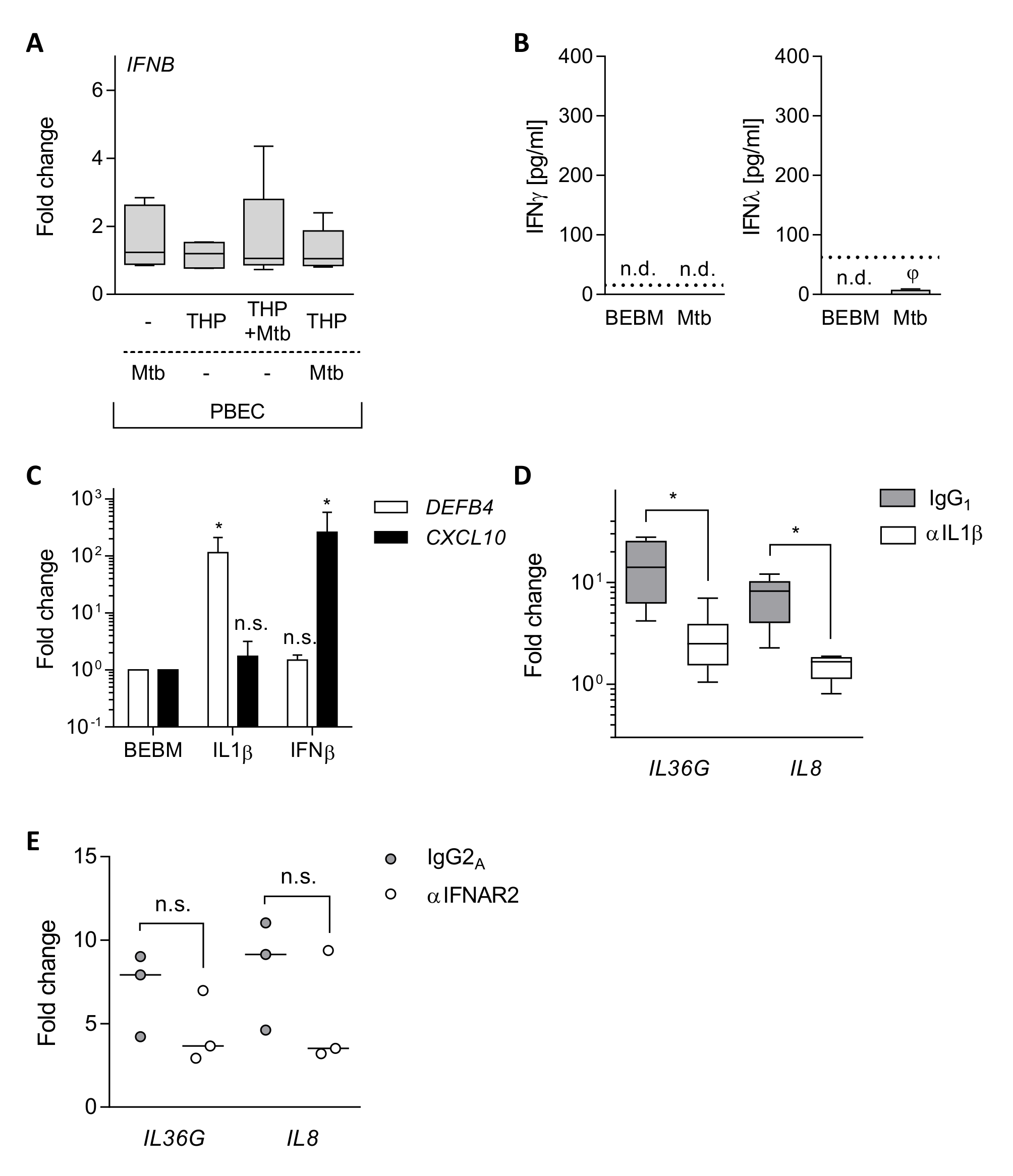

Supplement: S3 Fig — A)PBECs were stimulated in the transwell co-culture system as indicated and IFNB expression measured after 24h. Fold change was calculated over unstimulated cells. Boxplots show median and range (n = 5). No significant differences were detected.B)IFNγ and IFNλ release by THP-1 cells after 24h of Mtb-infection (MOI5) was measured by ELISA in two independent experiments. Mean ± SD. n.d., not detected; φ, extrapolated values below the detection limit; horizontal lines indicate the detection limits of the assays.C)PBECs were stimulated with 1 ng/ml IL1β or IFNβ for 24h and gene expression was measured by RT-PCR (n = 3). Mean ± SD are shown.D)PBECs were co-cultured with Mtb-infected THP-1 cells in the presence of 20 μg/ml αL1β or IgG1 (isotype control) as indicated. After 24h, gene expression was measured by RT-PCR. Expression is shown as fold change over unstimulated (n = 6). Boxplots show median and range.E)PBECs were co-cultured with Mtb-infected THP-1 cells in the presence of 20 μg/ml αIFNAR2 or IgG2 (isotype control). After 24h, gene expression was measured by RT-PCR and is shown as fold change over unstimulated (n = 3). Median is shown. Friedman test with Dunn’s post-test was used to compare groups against isotype control. n.s., not significant; *, p<0.05. (TIF) [file ppat.1006577.s003.tif]

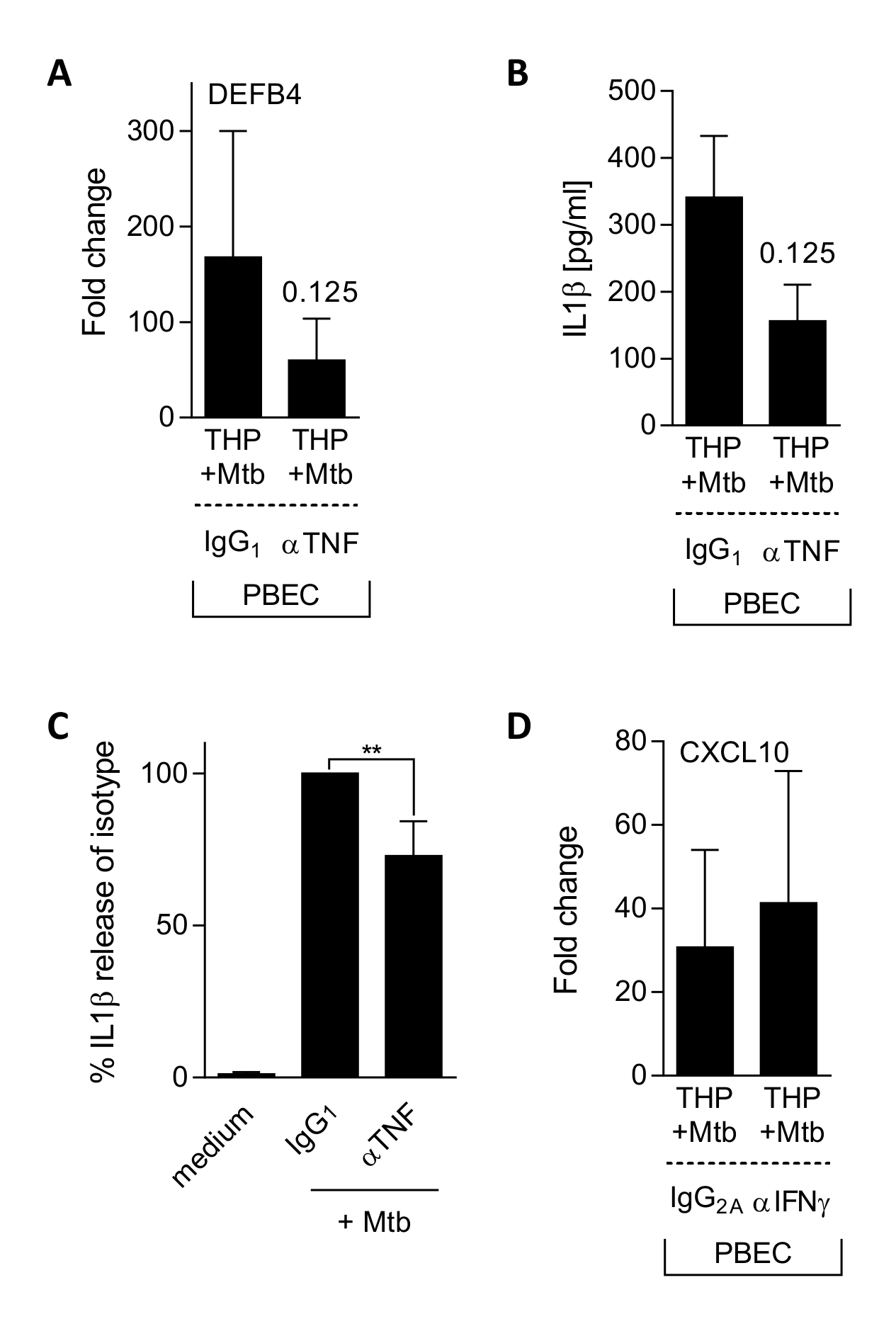

Supplement: S4 Fig — (A)PBECs were co-cultured with Mtb-infected (MOI5) THP-1 cells with αTNF or IgG1 for 24h. DEFB4 expression was measured by RT-PCR and is shown as fold change over unstimulated PBECs (n = 3).(B)IL1β release was measured in the co-culture supernatants of (A) by ELISA.(C)THP-1 MΦs were infected with Mtb (MOI5) in the presence of αTNF or IgG1 and IL1β release measured after 24h. Cytokine levels are shown as % of IL1 β release during infection in the presence of IgG1 (n = 5).(D)PBECs were exposed to Mtb-infected THP-1 cells (MOI5) in co-culture in the presence of αIFNγ or IgG2a. After 24h, CXCL10 expression was measured by RT-PCR and is shown as fold change over unstimulated PBECs. Mean ± SD are shown. (A, B and D) Wilcoxon signed rank test was used to compare groups; (C) was compared by repeat-measure ANOVA with Holm-Sidak's multiple comparisons test. **, p<0.01 or exact p-values are given. (TIF) [file ppat.1006577.s004.tif]

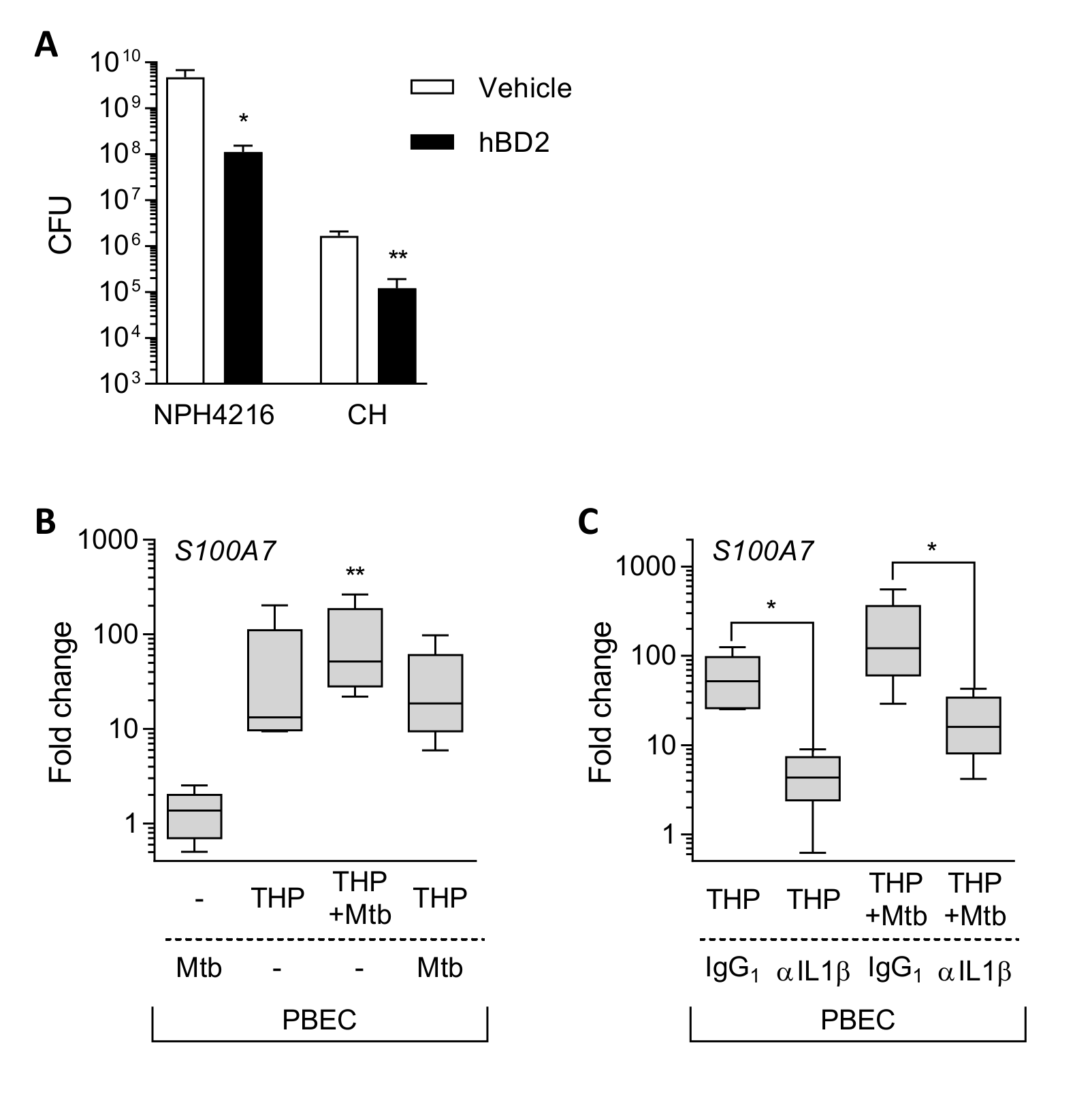

Supplement: S5 Fig — (A)Clinical isolates Mtb NPH4216 and Mtb CH were incubated with 5 μg/ml recombinant hBD2 or vehicle control as described in Fig 8. Colony forming units (CFU) were determined at day 7. Effects of hBD2 was compared with vehicle control by Student t-test. Mean ± SD of triplicate measurements are shown. * p<0.05; ** p<0.01(B)In the transwell model, PBECs were exposed to THP-1 cells or Mtb H37Rv (MOI5 over THP-1) for 24h as indicated. S100A7 expression in PBECs was measured by RT-PCR and is shown as fold change over unstimulated PBECs (n = 5).(C)PBECs were co-cultured with infected or uninfected THP-1 cells in the presence of αL1β or IgG1 as indicated. After 24h, S100A7 expression was measured by RT-PCR and is shown as fold change over unstimulated PBECs (n = 5). Friedman test with Dunn’s post-test was used to compare expression with unstimulated or respective isotype control. Boxplots show median and range. * p<0.05; ** p<0.01. (TIF) [file ppat.1006577.s005.tif]

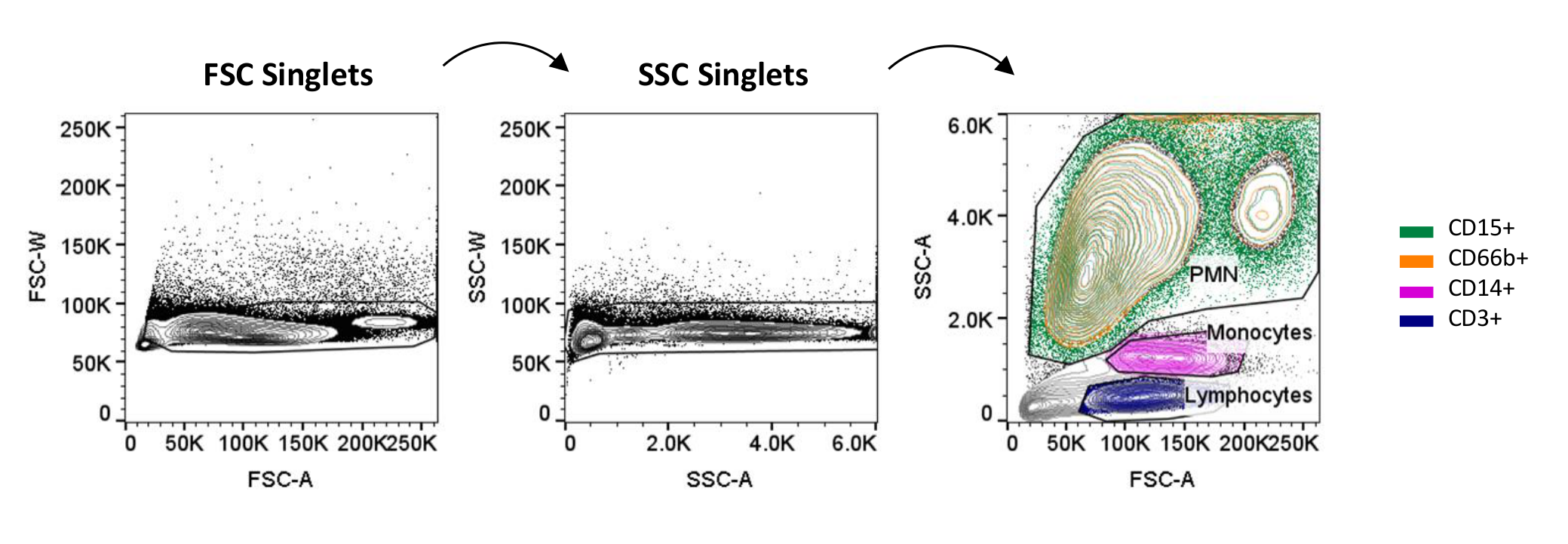

Supplement: S6 Fig — PBLs were isolated from whole blood and stained for CD3, CD14, CD15 and CD66b. Shown are representative plots for the gating strategy from one of three donors. After gating for singlets, forward (FSC) and side (SSC) scatter were used to define PBL subsets. PMN, polymorphonuclear cells. (TIF) [file ppat.1006577.s006.tif]
